# Supplementary material for: Molecular principles of redox-coupled sodium pumping of the ancient Rnf machinery
Source: Nat Commun. 2025 Mar 7;16:2302. doi: 10.1038/s41467-025-57375-8 (PMC11889175; doi:10.1038/s41467-025-57375-8)
Supplement: Supplementary file 1 — Supplementary Information [file 41467_2025_57375_MOESM1_ESM.pdf]

Molecular principles of redox-coupled sodium pumping of the ancient Rnf machinery

Anuj Kumar[1,2†], Jennifer Roth[2†], Hyunho Kim[3†], Patricia Saura[3†], Stefan Bohn[4], Tristan Reif-Trauttmansdorff[1], Anja Schubert[2], Ville R. I. Kaila[3*], Jan M. Schuller[1*], Volker Müller[2*]

[1]SYNMIKRO Research Center and Department of Chemistry, Philipps-University of Marburg, Marburg, Germany.
[2]Department of Molecular Microbiology & Bioenergetics, Institute of Molecular Biosciences, Johann Wolfgang Goethe University, Frankfurt am Main, Germany.
[3]Department of Biochemistry and Biophysics, Stockholm University, Stockholm, Sweden.
[4]Cryo-Electron Microscopy Platform and Institute of Structural Biology, Helmholtz Munich; Ingolstädter Landstraße 1, 85764 Neuherberg, Germany.

†Equal contribution
*Correspondence to: ville.kaila@dbb.su.se; jan.schuller@synmikro.uni-marburg.de; and vmueller@bio.uni-frankfurt.de

Content

Supplementary Methods
- Molecular dynamics simulations
- Steered molecular dynamics simulation of the *inward-outward* transition
- Free energy simulations using the string method
- Electrostatic calculations
- Analysis of Na^+ pathways and binding sites during MD simulations
- Free energy profiles for Na^+ transport
- Simulation of the Na^+ translocation kinetics

Supplementary Tables
Supplementary Table 1 | Ferredoxin:NAD^+ oxidoreductase activities of purified Rnf-variants.
Supplementary Table 2 | Determination of the iron content for the WT Rnf complex and its variants.
Supplementary Table 3 | Growth rates and doubling time.
Supplementary Table 4 | Cryo-EM data collection, refinement, and validation statistics.
Supplementary Table 5 | Comparison of residues in NqrB of the Nqr complex and in RnfD of the Rnf complex.
Supplementary Table 6 | List of atomistic MD simulations.
Supplementary Table 7 | Non-standard protonation states in the MD simulations.
Supplementary Table 8 | Estimation of electron transfer rates.
Supplementary Table 9 | Plasmids generated during this study.
Supplementary Table 10 | RESP charges of iron-sulphur clusters, FMN and riboflavin cofactors.

Supplementary Figures
Supplementary Fig. 1 | Purification and characterisation of the Rnf complex from *A. woodii*.
Supplementary Fig. 2 | Cryo-EM data collection and analysis of the Rnf complex with NADH bound.
Supplementary Fig. 3 | Cryo-EM data collection and analysis of the Rnf complex reduced with pre-reduced Fd.
Supplementary Fig. 4 | Cryo-EM data collection and analysis of the *apo* state of the Rnf complex.
Supplementary Fig. 5 | Cryo-EM density and model quality.
Supplementary Fig. 6 | Comparison of cofactor distances in different states of the Rnf complex resolved by cryo-EM
Supplementary Fig. 7 | Structural comparison of Rnf and Nqr and sequence conservation.
Supplementary Fig. 8 | MD simulations of the Rnf complex.
Supplementary Fig. 9 | Characterisation of the inward/outward conformations.
Supplementary Fig. 10 | Summary of distances between cofactors from MD simulations.
Supplementary Fig. 11 | Global dynamics of the Rnf complex from principal component analysis (PCA) of the MD simulations.
Supplementary Fig. 12 | Sodium binding in the membrane domain of Rnf.
Supplementary Fig. 13 | Kinetic simulations of the sodium translocation process.
Supplementary Fig. 14 | Purification and characterisation of Rnf variants from *A. woodii*.
Supplementary Fig. 15 | Sodium binding to the RnfA/E dimer.

Supplementary Fig. 16 | Overview of hydration in the membrane subunits of Rnf.
Supplementary Fig. 17 | Ion channel analysis in RnfA/E.
Supplementary Fig. 18 | Steered molecular dynamics exploration of the *inward/outward* transition.
Supplementary Fig. 19 | Free energy calculations of the *inward-outward* transition.
Supplementary Fig. 20 | Characterisation of structures and sampling along the string simulations.

Supplementary References

Supplementary Methods

Molecular dynamics simulations

Two different initial models were created starting from structures/cryo-EM density obtained from the NADH- or the ferredoxin (Fd)-reduced experimental conditions. For the NADH-reduced model, the resolved 3.3 Å cryo-EM structure (PDB ID: 9ERI) was embedded in a POPC membrane using CHARMM-GUI[1], and the system was hydrated in a TIP3P water box. Na^+ and Cl^- ions were added to neutralise the system with a salt concentration of 250 mM, higher than the physiological value (150 mM) to increase the likelihood of Na^+ binding to the protein. The Fd-reduced protein model was generated using molecular dynamics flexible fitting (MDFF)[2] by fitting the NADH-reduced structure to the density of the Fd-reduced Rnf using MDFF. To this end, the coordinates of the protein were relaxed to the cryo-EM map for 2 ns, with secondary structure restraints applied to protein residues. The protonation states of the protein sidechains and disulphide bridges were determined using PROPKA3 v. 3.4.0[3]. The FMN cofactors, located in RnfG and RnfD subunits were modelled by covalently linking them to $T185^{RnfG}$ and $T156^{RnfD}$ via a phosphodiester bond (Supplementary Fig. 8a). The full system comprised *ca.* 430,000 atoms, with the NADH-reduced model including 100,572 water molecules, 506/482 Na^+/Cl^- ions, and 448 POPC molecules, and the Fd-reduced model containing 115,908 water molecules, 566/553 Na^+/Cl^- ions, and 448 POPC molecules. The MD simulation box dimensions were 170x164x180 Å and 166x158x208 Å for the NADH- and Fd-reduced models, respectively. To probe the effect of cofactor redox state on protein conformation and sodium binding, the iron-sulphur clusters, FMN, or riboflavin cofactors were modelled in different redox states (see Supplementary Table 6). All simulations were performed using the CHARMM36 force field in combination with in-house DFT-based parameters for the cofactors[4–6] (see Supplementary Table 10). All simulations were run using NAMD[7] versions 2.14 or 3.0 in an *NPT* ensemble with Nosé-Hoover-Langevin pressure control (p=1 atm) and Langevin thermostat (T=310 K), with the integration timestep set to 2 fs and rigid bonds set for hydrogens using the shakeH algorithm. Long-range electrostatic interactions were implemented using the Particle-Mesh Ewald (PME) method with the grid spacing set to 1 Å, while the switching and cutoff distances for the Lennard-Jones potential were set to 10 and 12 Å, respectively. The MD trajectories were analysed using VMD[8] and MDAnalysis[9].

Steered molecular dynamics simulation of the inward/outward transition

The initial pathway of the inward/outward transition was explored by steered molecular dynamics simulations (SMD) using NAMD 3.0[7] together with the Colvars[10] module to define the collective variables (CV). To this end, a snapshot with a Na^+ ion bound in the intracellular buried site was selected from MD simulation S15 (see Supplementary Table 6). The conformation of the RnfA/E subunits, initially in the inward state, was biased towards the outward state by decreasing the R_1 CV, defined as the mean distance between group 1 (Cα atoms of residues $Q85^{RnfA}$ to $T110^{RnfA}$) and group 2 (Cα atoms of residues $C108^{RnfE}$ to $A118^{RnfE}$), located on the intracellular side, while increasing the mean distance (R_2) between group 3 (Cα atoms of residue $T80^{RnfE}$ to $V105^{RnfE}$) and group 4 (Cα atoms of residues $L116^{RnfA}$ to $E126^{RnfA}$) on the extracellular side (see Supplementary Fig. 18a for a representation of the CVs). The CVs were biased by applying harmonic restraints with a force constant of 200 kcal mol^{-1} $Å^{-2}$ for R_1 and R_2. During the SMD simulations, R_1 was linearly decreased from 17.7 Å to 10.8 Å, while R_2 was increased from 14.2 Å to 21 Å over 195 ns, respectively. The MD setup and force field employed were the same as in the unbiased MD simulations (see above). The trajectories were analysed using VMD[8] and MDAnalysis[9].

Free energy simulations using the string method

To optimise the reaction pathway of the inward/outward conformational transition coupled to Na^+ ion transport, we employed an in-house implementation of the finite temperature string method[11,12], adapted into NAMD. The conformational transition was explored by a 2D reaction coordinate comprising RC_1 and RC_2, which describe the inward to outward conformational transition of the RnfA/E subunits and Na^+ transport across it, respectively. Specifically, RC_1 was defined as R_1 - R_2, where R_1 is the mean

distance between group 1 (Cα atoms of residues $Q85^{RnfA}$ to $T110^{RnfA}$) and group 2 (Cα atoms of residues $C108^{RnfE}$ to $A118^{RnfE}$), and R_2, is the mean distance between group 3 (Cα atoms of residue $T80^{RnfE}$ to $V105^{RnfE}$) and group 4 (Cα atoms of residues $L116^{RnfA}$ to $E126^{RnfA}$, see Supplementary Fig. 19a). RC_2 was defined as the distance between the Na^+ ion and the RnfA/E centre of mass projected along the Z-axis (Supplementary Fig. 19b). The initial pathway was generated by interpolating equidistant points between the inward/Na^+-cytosolic state (RC_1 = -6 Å and RC_2 = 4 Å) and the outward/Na^+-extracellular states (RC_1 = 9 Å and RC_2 = -4 Å) (Supplementary Fig. 19d) with 15 equidistant windows created along the pathway. In this regard, the structures were extracted by projection of the RC_1 dimension onto the trajectory sampled by the SMD simulations (see above, Supplementary Fig. 19c). The parallel windows were restrained by harmonic potentials on both RC_1 and RC_2 with force constants ranging from 10 to 25 kcal mol^{-1} $Å^{-2}$ and sampled for 10 ns per window for each string iteration (Supplementary Fig. 19d,g). A new string pathway was obtained by fitting the string using splines, followed by equal separation of the new windows along the string for the next iteration. The convergence of the string optimisation was evaluated by computing the sum of squares and maximum deviation between each string iteration (Supplementary Fig. 19j,k), and set to 0.008 $Å^2$ and 0.05 Å, respectively. The string optimisation was performed for both the reduced and oxidised form of the AE1-cluster. The oxidised state simulations were started from the 5th iteration of the string optimisation of the reduced state (see Supplementary Fig. 19d,g). The converged string pathway were further extended by placing 30 equally spaced windows along the optimised string, and subjected to 45 ns sampling with the force constants set to 15 kcal mol^{-1} $Å^{-2}$ on RC_1 and RC_2, with 5 windows further added for the reduced state to fully sample the phase space around the reactant minimum. The final potential of mean force was calculated using 2D-WHAM[13]. The trajectories were analysed using VMD[8] and MDAnalysis[9].

Electrostatic calculations

Sodium binding energies were estimated using a Molecular Mechanics Poisson-Boltzmann Surface Area (PBSA/MM) model as implemented in APBS[14,15]. In this regard, MD snapshots with sodium bound next to the AE1 FeS centre were selected, with a cutoff of 7 Å, from simulations S15-S18 (Supplementary Table 6). The protein subunits RnfA, RnfE, the AE1 FeS centre, and the bound sodium were included in the models for estimation of the interaction energies. The solvent was modelled with a dielectric constant of 78.4, the NaCl concentration set to 250 mM, whilst the protein interior was modelled as a polarizable dielectric medium with an $\varepsilon=4$. The interactions between protein residues and sodium were described by explicit atomic point charges and Lennard-Jones interactions.

Analysis of Na^+ pathways and binding sites during MD simulations

A potential tunnel pathway through the RnfA/E subunits was calculated using CAVER[16]. Structural snapshots with only the protein were extracted from MD simulations S15 (inward) and S16 (outward) (Supplementary Fig. 17a-d). In this regard, the starting point was set to search pathways between residues $C25^{RnfA}$ and $C108^{RnfE}$. The probe radius, shell radius, and shell depth were set to 0.8 Å, 3.4 Å, and 3.5 Å, respectively. For analysing the Na^+ ion pathway during the last 100 ns of MD simulations S15 (inward) and S16 (outward) AQUA-DUCT[17] was used (Supplementary Fig. 17e,f). Here, the pathway search was extended to the whole protein with aims to track any Na^+ ions entering within 10 Å radius of the AE1 cluster. The resulting data was analysed using PyMol 2.3.0[18].

Free energy profiles for Na^+ transport

Free energy profiles for the redox-driven Na^+ pumping in Rnf were derived based on redox potentials for Fd (-450 mV), FeS (-320 mV), FMN (-280 mV), RFB (-230 mV), and NAD^+ (-320 mV). The Na^+ affinity for the reduced AE1 centre was -130 mV (-3.1 kcal mol^{-1}), obtained from PBSA/MM calculations, which was also used to tune the redox potential of the AE1 centre to -190 mV in the Na^+ bound state to fulfil microscopic reversibility/detailed balance. The effect of the SMF on the free energy levels were divided equally based on the relative location of the cofactors in the membrane plane, $(z/L)\Delta\psi$, with the membrane thickness L (32 Å) and the SMF ($\Delta\psi$) set to 180 mV. This ansatz yielded

shifts in the electron transfer reactions for B8 → AE1 (+0.375L, $\Delta\Delta G$=-67.5 mV); AE1 → FMN^G (+0.375L, $\Delta\Delta G$=-67.5 mV); FMN^G → FMN^D (-0.125L, $\Delta\Delta G$=+22.5 mV); FMN^D→ RBF (-0.25L, $\Delta\Delta G$=+45 mV); and RBF→ C1 (-0.375L, $\Delta\Delta G$= +67.5 mV), with the positive (+) directions defined from the N-side → P-side. Moreover, transferring the Na^+ ion from the inside to AE1 (+0.375L) and from AE1 to the cytoplasmic side (+0.625L) increased the free energy of the respective states by +67.5 mV and +112.5 mV at a 180 mV SMF.

The barriers for the electron transfer reactions were computed based on electron transfer theory[19] using the Moser-Dutton model[20],

$$\log k = 13 - (1.2-0.8\rho)\ (r-3.6) - 3.1(\Delta G+\lambda)^2 / \lambda \quad (1a)$$

and for reactions with $\Delta G > 0$[20],

$$\log k = 13 - (1.2-0.8\rho)\ (r-3.6) - 3.1(-\Delta G+\lambda)^2 / \lambda - \Delta G/0.06 \quad (1b)$$

where ρ is the protein packing density (here assumed 0.76[20,21]), r is the edge-to-edges distance, ΔG is the driving force for the electron transfer reaction, and λ is the reorganisation energy (0.7 eV[20]). The electron transfer rates were converted into activation free energies ($\Delta G^{\ddagger}$) using transition state theory,

$$k = \kappa\ (k_B T/h)\ \exp(-\Delta G^{\ddagger}/RT) \quad (2)$$

with κ=1, and $k_B T/h \sim 6.45\ ps^{-1}$. The rate of sodium binding to the reduced active AE1 site was estimated based on MD simulations to ca. 0.5 µs ($\Delta G^{\ddagger}$~0.4 eV).

Simulation of the Na^+ translocation kinetics

Kinetic simulations were performed by numerical integration of the master-equation,

$$\frac{dp_i}{dt} = \sum_j k_{ji} p_j - \sum_j k_{ij} p_i \quad (3)$$

for all transitions between energy levels i and j with forward (k_{ij}) and backward (k_{ij}) rates derived from the free energy profiles (Fig. 4b). The initial/final electron transfer steps were modeled as irreversible. The kinetic simulations were performed using COPASI[22]. See Supplementary Fig. 13 for further details on the kinetic simulations.

Supplementary Tables

Supplementary Table 1 | Ferredoxin:NAD^+ oxidoreductase activities of purified Rnf-variants. Activities are the mean of three independent biological replicates, measured in triplicates (n = 3).

Variant	Fd_{red}:NAD^+ [U mg^{-1}]
**Rnf complementation**	7.1 ± 1.1
**RnfΔAE1**	-
**RnfΔB1**	-
**RnfD D249A/N123A**	-
**RnfG T185A/Y113A**	-
**RnfA Y105A**	3.4 ± 0.7
**RnfE R67A**	0.7 ± 0.2
**RnfE L103G**	0.8 ± 0.2

Supplementary Table 2 | Determination of the iron content for the WT Rnf complex and its variants. The measurements are the mean of three independent biological replicates, measured in triplicates (n = 3).

Variant	Mol Fe/mol Rnf
**Rnf complementation**	41.8 ± 1.5
**RnfΔAE1**	40.4 ± 1.1
**RnfΔB1**	38.4 ± 2.2
**RnfD D249A/N123A**	41.9 ± 2.0
**RnfG T185A/Y113A**	42.3 ± 1.7
**RnfA Y105A**	42.1 ± 2.3
**RnfE R67A**	42.2 ± 1.9
**RnfE L103G**	42.9 ± 2.3

Supplementary Table 3 | Growth rates and doubling time. Data was obtained after growth on $H_2 + CO_2$ for ~ 90 h in complex medium containing 20 mM of NaCl. Values are the mean of three independent biological replicates, measured in triplicates (n = 3).

Variant	final OD_{600}	Growth rate μ [h^{-1}]	Doubling time [h]
ΔpyrE	0.477 ± 0.05	0.02	28 ± 1.7
Δrnf	n.g.[1]	n.g.[1]	n.g.[1]
**Rnf complementation**	0.453 ± 0.02	0.03	30 ± 2.0
**RnfΔAE1**	n.g.[1]	n.g.[1]	n.g.[1]
**RnfΔB1**	n.g.[1]	n.g.[1]	n.g.[1]
**RnfD D249A/N123A**	n.g.[1]	n.g.[1]	n.g.[1]
**RnfG T185A/Y113A**	n.g.[1]	n.g.[1]	n.g.[1]
**RnfA Q85A**	0.276 ± 0.02	0.015	43.5 ± 2.0
**RnfA Y105A**	0.175 ± 0.03	0.01	58.5 ± 2.5
**RnfA T110G**	n.g.[1]	n.g.[1]	n.g.[1]
**RnfA T111G**	0.411 ± 0.04	0.03	31.2 ± 1.3
**RnfE R67A**	n.g.[1]	n.g.[1]	n.g.[1]
**RnfE L103G**	n.g.[1]	n.g.[1]	n.g.[1]
**RnfE V106G**	n.g.[1]	n.g.[1]	n.g.[1]
**RnfE N107A**	0.150 ± 0.05	0.007	75.2 ± 3.1
**RnfE E115A**	0.390 ± 0.04	0.03	34.4 ± 2.2
**RnfE E115Q**	0.200 ± 0.05	0.01	65.3 ± 3.9
**RnfE E115K**	n.g.[1]	n.g.[1]	n.g.[1]
**RnfD I274A**	0.479 ± 0.07	0.03	31.2 ± 3.0
**RnfD R275A**	0.449 ± 0.03	0.02	30.7 ± 2.8
**RnfD Y280A**	0.440 ± 0.02	0.03	33.1 ± 4.0
**RnfD L241A**	0.457 ± 0.02	0.03	32.4 ± 3.5
**RnfD F245A**	0.455 ± 0.03	0.02	29.5 ± 3.0

[1]n. g., no growth was observed

Supplementary Table 4 | Cryo-EM data collection, refinement, and validation statistics.

	**Rnf NADH bound state** (EMDB - 19915) (PDB - 9ERI)	**Rnf Fd-reduced State-1 (consensus map)** (EMDB - 19919) (PDB - 9ERK)	**Rnf Fd-reduced State-2 (B8 closer to membrane)** (EMDB - 19916) (PDB - 9ERJ)	**Rnf *apo* state** (EMDB - 19920) (PDB - 9ERL)
**Data collection and processing**				
Magnification	60,000 x[a]	165,000 x[b]	165,000 x[b]	165,000 x[b]
Voltage (kV)	300	300	300	300
Electron exposure (e–/Å^2)	50.0	50.0	50.0	50.0
Defocus range (μm)	0.8-1.8	1.2-1.8	1.2-1.8	1.2-1.8
Pixel size (Å)	1.09	0.75	0.75	0.84
Symmetry imposed	C1	C1	C1	C1
Initial particle images (no.)	3622306	5365845	5365845	2513935
Final particle images (no.)	645102	604534	260238	251476
Map resolution (Å)	3.3	2.8	2.99	3.0
FSC threshold	0.143	0.143	0.143	0.143
Map resolution range (Å)	3.0-4.5	2.5-4.0	2.8-4.0	2.8-4.0
**Refinement**				
Initial model used (PDB code)	*de novo,* AlphaFold	*de novo*, AlphaFold	*de novo*, AlphaFold	*de novo*, AlphaFold
Model resolution (Å)	3.4	3.0	3.2	3.2
FSC threshold	0.5	0.5	0.5	0.5
Model resolution range (Å)	3.3-3.6	2.8-3.0	2.9-3.2	3.2-3.6
Map sharpening *B* factor (Å^2)	-209.8	-132.6	-110.9	-112.7
Model composition				
Non-hydrogen atoms	12706	12669	12665	12665
Protein residues	1688	1688	1688	1688
Ligands	FMN: 3, NADH: 1, RBF: 1, FES: 1, SF4: 10	FMN: 3, NADH: 0, RBF: 1, FES: 1, SF4: 10, Na: 7	FMN: 3, NADH: 0, RBF: 1, FES: 1, SF4: 10, Na: 3	FMN: 3, NADH: 0, RBF: 1, FES: 1, SF4: 10, Na: 3
B factors (Å^2)				
Protein	47.73	31.78	38.81	12.80
Ligand	50.96	51.81	70.93	22.86
R.m.s. deviations				
Bond lengths (Å)	0.005	0.007	0.007	0.011
Bond angles (°)	0.778	1.159	1.097	1.231
Validation				
MolProbity score	1.72	1.64	1.62	1.61
Clashscore	5.40	3.62	3.47	4.01
Poor rotamers (%)	0.58	0.52	0.45	0.67
Ramachandran plot				
Favored (%)	96.44	96.00	96.24	95.50
Allowed (%)	3.02	3.28	3.22	4.08
Disallowed (%)	0.54	0.72	0.54	0.42

a: Data acquired on a Krios 1/ K3 direct electron detector camera, pixel size = 1.09 Å.
b: Data acquired on a Krios G4/ Falcon 4i camera, pixel size = 0.76 Å.

Supplementary Table 5 | Comparison of residues present in NqrB of the Nqr complex and in RnfD of the Rnf complex. Residues in NqrB involved in sodium translocation and the corresponding residues in RnfD are indicated. Conserved residues in Na^{+}- and H^{+} -dependent Rnfs are highlighted. Sequence alignments were done with ClustalOmega[23].

**Residue in NqrB**	**Residue in RnfD**	**Conserved residue in Na^{+}- and H^{+} -dependent Rnfs**
**Na-1**		
A263	T177	-[1]
V275	I189	-[1]
V332	V235	-[1]
**Na-2**		
I371	I274	yes
R372	R275	yes
P376	-[1]	
Y378	Y280	yes
F338	L241	yes
F342	F245	yes

[1]-: residue not present in the sequence or not conserved in Na^{+}- and H^{+} -dependent Rnfs

Supplementary Table 6 | List of atomistic MD simulations. The cumulative MD sampling was 16.8 μs. [a] Model generated based on initial MDFF relaxation. [b] See Supplementary Fig. 9 for classification of conformation.

**Simulation**	**PDB**	**Substrate**	**Reduced cofactor**	**RnfA/E conformation**[b]	**Length (ns)**
S0	9ERI (nadh)	NAD^+	-	outward	700
S1	9ERI (nadh)	NAD^+	B8	outward	500
S2	9ERI (nadh)	NAD^+	B8	outward	500
S3	9ERI (nadh)	NAD^+	AE1	outward	500
S4	9ERI (nadh)	NAD^+	AE1	outward	500
S5	9ERI (nadh)	NAD^+	B8, AE1	outward	500
S6	9ERI (nadh)	NAD^+	B8, AE1	outward	500
S7	9ERI (nadh)	NAD^+	B7, B8	outward	400
S8	9ERI (nadh)	NAD^+	B7, B8	outward	400
S9	9ERI (nadh)	NAD^+	AE1, FMN^G	outward	300
S10	9ERI (nadh)	NAD^+	AE1, FMN^G	outward	300
S11	9ERI (nadh)	NAD^+	FMNG, FMN^D	outward	500
S12	9ERI (nadh)	NAD^+	FMNG, FMN^D	outward	500
S13	9ERJ (fd)[a]	-	B7, B8	outward	1000
S14	9ERJ (fd)	-	B7, B8	inward	1000
S15	9ERJ (fd)	-	B8, AE1	inward	1000
S16	9ERJ (fd)	-	B8, AE1	outward	1000
S17	9ERJ (fd)	-	AE1, FMN^G	outward	1000
S18	9ERJ (fd)	-	AE1, FMN^G	inward	1000
S19	9ERJ (fd)	-	FMN^G, FMN^D	inward	1000
S20	9ERJ (fd)	-	FMN^G, FMN^D	inward	1000
S21	9ERJ (fd)	-	B8, FMN^G	inward	200
S22	9ERJ (fd)	-	B8, FMN^G	outward	200
S23	9ERJ (fd)	-	B8, FMN^D	inward	200
S24	9ERJ (fd)	-	B8, FMN^D	inward	200
S25	9ERJ (fd)	-	B8, RBF	inward	200
S26	9ERJ (fd)	-	B8, RBF	inward	200
S27	9ERJ (fd)	-	RBF, FMN^D	inward	500
S28	9ERJ (fd)	-	RBF, FMN^D	outward	500
Total					16.8 μs

Supplementary Table 7 | Non-standard protonation states in the MD simulations. The protonation states were determined using PROPKA3[3] calculation based on the NADH-reduced cryo-EM structure. (ε) - ε protonated (neutral) histidine, (δ) - δ protonated (neutral) histidine, (ε/δ) - positively charged doubled protonated histidine (HisH$^+$). Unless stated otherwise, histidine residues were modelled in δ-protonated state.

**Subunit**	**Residues**
RnfA	-
RnfB	E127
RnfC	E167, E169, H5(ε/δ)
RnfD	E61, E188, D249, H139(ε/δ)
RnfE	-
RnfG	E87, E139

Supplementary Table 8 | Estimation of electron transfer rates. Redox potentials: Fd (-450 mV), FeS (-320 mV), FMN (-280 mV), RFB (-230 mV), NAD^+ (-320 mV) [a] State is not sampled. <*r*> – average *edge-to-edge* distances from MD simulations; the λ – reorganisation energy was modelled as 0.7 eV[20]; ρ – protein packing density was set to 0.76[20]; $\Delta G^‡$ – Activation free energies based on transition state theory from k_{eT}, with standard pre-exponential factors and κ=1; electron transfer rate k_{eT} from Moser-Dutton model[20] (see also Ref. [21]). [c] shortest distance from MD snapshots. [d] based on FMN/NADH PCET reaction in Complex I, ca. 10,000 s^{-1} [24].

D	A	State	<*r* (Å)>	ΔG (eV)	$\Delta G^‡$ (eV)	t_{eT} (s)	ΔG (eV) SMF	$\Delta G^‡$ (eV) SMF	t_{eT} (s) SMF
Fd	B1	N/A[a]	14[a]	-0.13	0.46	3.9×10^{-6}	-0.13	0.46	3.9×10^{-6}
B1	B2	inward	9.6	0	0.34	5.3×10^{-8}	0	0.34	5.3×10^{-8}
B1	B2	outward	11.7	0	0.29	9.2×10^{-9}	0	0.29	9.2×10^{-9}
B2	B3	inward	13.0	0	0.28	5.4×10^{-9}	0	0.28	5.4×10^{-9}
B2	B3	outward	16.3	0	0.58	0.5×10^{-3}	0	0.58	0.5×10^{-3}
B3	B4	inward	11.1	0	0.40	4.1×10^{-7}	0	0.40	4.1×10^{-7}
B3	B4	outward	10.6	0	0.38	2.1×10^{-7}	0	0.38	2.1×10^{-7}
B4	B5	inward	11.3	0	0.40	5.3×10^{-7}	0	0.40	5.3×10^{-7}
B4	B5	outward	11.2	0	0.40	4.7×10^{-7}	0	0.40	4.7×10^{-7}
B5	B6	inward	10.5	0	0.37	1.8×10^{-7}	0	0.37	1.8×10^{-7}
B5	B6	outward	10.8	0	0.38	2.7×10^{-7}	0	0.38	2.7×10^{-7}
B6	B7	inward	10.9	0	0.39	3.1×10^{-7}	0	0.39	3.1×10^{-7}
B6	B7	outward	11.3	0	0.40	5.4×10^{-7}	0	0.40	5.4×10^{-7}
B7	B8	inward	11.0	0	0.39	3.6×10^{-7}	0	0.39	3.6×10^{-7}
B7	B8	outward	9.1	0	0.32	2.7×10^{-8}	0	0.32	2.7×10^{-8}
B8	AE1	inward	15[c]	0	0.54	8.3×10^{-5}	-0.07	0.51	3.2×10^{-5}
B8	AE1	inward + Na^+	15[c]	-0.26	0.46	4.0×10^{-6}	-0.13	0.49	1.5×10^{-5}
B8	AE1	outward	20.7	0	0.74	0.2	-0.07	0.72	0.08
B8	AE1	outward + Na^+	20.7	-0.26	0.66	0.01	-0.13	0.70	0.04
AE1	FMN^G	inward	23.1	+0.09	0.87	20.3	+0.13	0.89	39.4
AE1	FMN^G	outward	15.1	+0.09	0.58	0.4×10^{-3}	+0.13	0.59	0.7×10^{-3}
FMN^G	FMN^D	inward	11.6	0	0.41	8.1×10^{-7}	+0.03	0.42	1.2×10^{-6}
FMN^G	FMN^D	outward	12.1	0	0.43	1.6×10^{-6}	+0.03	0.44	2.5×10^{-6}
FMN^D	RBF	inward	10.6	-0.05	0.36	1.0×10^{-7}	0	0.38	2.1×10^{-7}
FMN^D	RBF	outward	10.9	-0.05	0.37	1.6×10^{-7}	0	0.39	3.1×10^{-7}
RBF	C1	inward	11.3	+0.09	0.44	2.1×10^{-6}	+0.1	0.44	2.5×10^{-6}
RBF	C1	outward	11.4	+0.09	0.44	2.4×10^{-6}	+0.1	0.45	2.8×10^{-6}
C1	C2	inward	9.4	0	0.33	4.0×10^{-8}	0	0.33	4.0×10^{-8}
C1	C2	outward	9.6	0	0.34	5.3×10^{-8}	0	0.34	5.3×10^{-8}
C1	FMN	inward	8.5	+0.04	0.32	2.1×10^{-8}	+0.04	0.32	2.1×10^{-8}
C1	FMN	outward	8.5	+0.04	0.32	2.1×10^{-8}	+0.04	0.32	2.1×10^{-8}
FMN	NAD^+	inward	**3.5**	-0.04	0.54	0.1×10^{-3}	-0.04	0.54	0.1×10^{-3}
FMN	NAD^+	outward	**3.5**	-0.04	0.54	0.1×10^{-3}	-0.04	0.54	0.1×10^{-3}

Supplementary Table 9 | Plasmids generated during this study.

Plasmid	Description	Number of oligonucleotides used[1]	Introduced mutations
pMTL84211_pPta_ack_ RnfCDGEAB (RnfG-His)	WT Rnf, C-terminal His-tag fused to RnfG	1+2+3+4+5+6	None
pMTL84211_pPta_ack_Rnf ΔAE1	Deletion of FeS cluster AE1	7+8+9+10+ 11+12+13+14	A-C25A, A-C113A, E-C25A, E-C108A
pMTL84211_pPta_ack_Rnf ΔB1	Deletion of FeS cluster B1	15+16	C50A, C53A, C58A, C75A
pMTL84211_pPta_ack_Rnf D D249A/N123A	Exchange of Riboflavin-binding D249 and N123 to A249 and A123 in RnfD	17+18+19+20	D249A, N123A
pMTL84211_pPta_ack_ RnfG T185A/Y113A	Exchange of FMN-binding T185 and Y113 to A185 and A113 in RnfG	21+22+23+24	T185A, Y113A
pMTL84211_pPta_ack_Rnf A Y105A	Exchange of Y105 to A in RnfA	25+26	Y105A
pMTL84211_pPta_ack_Rnf A T110G	Exchange of T110 to G in RnfA	27+28	T110G
pMTL84211_pPta_ack_Rnf A T111G	Exchange of T111 to G in RnfA	29+30	T111G
pMTL84211_pPta_ack_Rnf A Q85A	Exchange of Q85 to A in RnfA	31+32	Q85A
pMTL84211_pPta_ack_Rnf E R67A	Exchange of R67 to A in RnfE	33+34	R67A
pMTL84211_pPta_ack_Rnf E L103G	Exchange of L103 to G in RnfE	35+36	L103G
pMTL84211_pPta_ack_Rnf E V106G	Exchange of V106 to G in RnfE	37+38	V106G
pMTL84211_pPta_ack_Rnf E N107A	Exchange of N107 to A in RnfE	39+40	N107A
pMTL84211_pPta_ack_Rnf E E115A	Exchange of E115 to A in RnfE	41+42	E115A
pMTL84211_pPta_ack_Rnf E E115Q	Exchange of E115 to Q in RnfE	43+44	E115Q
pMTL84211_pPta_ack_Rnf E E115K	Exchange of E115 to K in RnfE	45+46	E115K
pMTL84211_pPta_ack_Rnf D I274A	Exchange of I274 to A in RnfD	47+48	I274A
pMTL84211_pPta_ack_Rnf D R275A	Exchange of R275 to A in RnfD	49+50	R275A
pMTL84211_pPta_ack_Rnf D Y280A	Exchange of Y280 to A in RnfD	51+52	Y280A
pMTL84211_pPta_ack_Rnf D L241A	Exchange of L241 to A in RnfD	53+54	L241A
pMTL84211_pPta_ack_Rnf D F245A	Exchange of F245 to A in RnfD	55+56	F245A

Supplementary Table 10 | RESP charges of iron-sulphur clusters, FMN and riboflavin cofactors. Four different FMN redox states are shown; FMN (oxidised, total charge = -2), $FMN^{-/\bullet}$ (reduced, $1e^-$, total charge = -3), $FMNH^{\bullet}$ (reduced, $1H^+/1e^-$, N5 protonated, total charge = -2), and $FMNH^-$ (reduced, $1H^+/2e^-$, N5 protonated, total charge = -3). Five different riboflavin (RBF) redox states are shown; RBF (oxidised, total charge = 0), $RBF^{-/\bullet}$ (reduced, $1e^-$ total charge = -1), $RBFH^{\bullet}$ (reduced, $1H^+/1e^-$, N5 protonated, total charge = 0), $RBFH^-$ (reduced, $1H^+$/2e-), $RBFH_2$ (reduced, $2H^+/2e^-$, N2 and N5 protonated, total charge = 0). RBFR bonded terms are from CHARMM36 cgenff parameters, while the charges were calculated using restrained electrostatic potential (RESP) at B3LYP-D3 / def2-TZVP/ ε = 4 level.

**4Fe4S**			**2Fe2S**		
**State**	**Oxidised 1FeIII/3FeIII**	**Reduced 2FeII/2FeIII**	**State**	**Oxidised FeIII/FeIII**	**Reduced FeII/FeIII**
**Atom Name**	**Charge**		**Atom Name**	**Charge**	
1FE1	0.797136	0.900785	1FE1	0.68059	0.74897
1FE2	0.761617	0.888277	1FE2	0.679	0.72132
1FE3	0.761617	0.900785	1S1	-0.66354	-0.84011
1FE4	0.797136	0.900785	1S2	-0.66342	-0.83904
1S1	-0.701499	-0.876966	2SG	-0.47826	-0.57245
1S2	-0.734161	-0.911752	2CB	-0.21208	-0.27218
1S3	-0.701499	-0.9968	2HB1	0.09	0.09
1S4	-0.734161	-0.891421	2HB2	0.09	0.09
2CB	0.091698	0.059323	3SG	-0.48211	-0.57436
2HB1	0.008776	0.009171	3CB	-0.20433	-0.27248
2HB2	0.008776	0.009171	3HB1	0.09	0.09
2SG	-0.62386	-0.822357	3HB2	0.09	0.09
3CB	0.091698	0.059324	4SG	-0.4788	-0.60735
3HB1	0.0115	0.002238	4CB	-0.21071	-0.3046
3HB2	0.0115	0.002238	4HB1	0.09	0.09
3SG	-0.62386	-0.822357	4HB2	0.09	0.09
4CB	0.100701	0.046293	5SG	-0.48335	-0.60812
4HB1	0.002629	0.001192	5CB	-0.203	-0.29959
4HB2	0.002629	0.001192	5HB1	0.09	0.09
4SG	-0.715789	-0.713624	5HB2	0.09	0.09
5CB	0.100701	0.059323			
5HB1	0.001252	0.008768			
5HB2	0.001252	0.008768			
5SG	-0.715789	-0.822357			

Supplementary Table 10 (continued).

FMN				
**State**	FMNO (FMN)	FMN1 ($FMN^{-/\bullet}$)	FMN2 ($FMNH^{\bullet}$)	FMN3 ($FMNH^{-}$)
**Atom Name**	**Charge**			
N1	-0.615943	-0.639088	-0.638309	-0.626021
C2	0.832762	0.81398	0.806648	0.691957
O2	-0.561696	-0.676036	-0.574344	-0.665953
N3	-0.608314	-0.593315	-0.561212	-0.561467
H3N	0.350301	0.313703	0.34164	0.318953
C4	0.555019	0.534704	0.576869	0.567696
O4	-0.507777	-0.602548	-0.561182	-0.671448
C4A	0.346809	0.228368	-0.142766	-0.189491
N5	-0.581155	-0.677167	-0.189397	-0.539849
H5N	-	-	0.304307	0.37613
C5A	0.519353	0.552962	0.156402	0.25892
C6	-0.429232	-0.498495	-0.453883	-0.511598
H6A	0.214339	0.19076	0.212203	0.187579
C7	0.195042	0.217973	0.221961	0.212641
C7M	-0.27782	-0.371534	-0.290236	-0.372657
H7M1	0.09	0.09	0.09	0.09
H7M2	0.09	0.09	0.09	0.09
H7M3	0.09	0.09	0.09	0.09
C8	0.109434	0.120895	0.117511	0.099859
C8M	-0.261718	-0.377383	-0.280291	-0.374304
H8M1	0.09	0.09	0.09	0.09
H8M2	0.09	0.09	0.09	0.09
H8M3	0.09	0.09	0.09	0.09
C9	-0.29736	-0.347211	-0.352379	-0.449271
H9	0.185945	0.176699	0.194833	0.204074
C9A	-0.194394	-0.286893	0.046639	0.174393
N10	0.24429	0.356739	0.052737	-0.133253
C10	0.112132	0.032016	0.349323	0.44234
C1'	-0.050017	-0.189131	-0.057074	-0.159231
H11	0.09	0.09	0.09	0.09
H12	0.09	0.09	0.09	0.09
C2'	0.191135	0.191135	0.191135	0.191135
H21	0.099466	0.099466	0.099466	0.099466
O2'	-0.652243	-0.652243	-0.652243	-0.652243
HO2'	0.412463	0.412463	0.412463	0.412463
C3'	0.092714	0.092714	0.092714	0.092714
H31	0.060543	0.060543	0.060543	0.060543
O3'	-0.660049	-0.660049	-0.660049	-0.660049
HO3'	0.384756	0.384756	0.384756	0.384756
C4'	0.26425	0.26425	0.26425	0.26425

H41	0.042698	0.042698	0.042698	0.042698
O4'	-0.745438	-0.745438	-0.745438	-0.745438
HO4'	0.446446	0.446446	0.446446	0.446446
C5'	0.202588	0.202588	0.202588	0.202588
H51	-0.078143	-0.078143	-0.078143	-0.078143
H52	-0.078143	-0.078143	-0.078143	-0.078143
O5'	-0.514227	-0.514227	-0.514227	-0.514227
P	1.247152	1.247152	1.247152	1.247152
O1P	-0.905323	-0.905323	-0.905323	-0.905323
O2P	-0.905323	-0.905323	-0.905323	-0.905323
O3P	-0.905323	-0.905323	-0.905323	-0.905323

Supplementary Table 10 (continued)

Riboflavin					
**State**	RBFO (RBF)	RBF1 ($RBF^{-/\bullet}$)	RBF2 ($RBFH^{\bullet}$)	RBF3 ($RBFH^{-}$)	RBFR ($RBFH_2$)
**Atom Name**	**Charge**				
N1	-0.615943	-0.639088	-0.638309	-0.626021	-0.446
H1					0.344
C2	0.832762	0.81398	0.806648	0.691957	0.474
O2	-0.561696	-0.676036	-0.574344	-0.665953	-0.504
N3	-0.608314	-0.593315	-0.561212	-0.561467	-0.415
H3	0.350301	0.313703	0.34164	0.318953	0.332
C4	0.555019	0.534704	0.576869	0.567696	0.551
O4	-0.507777	-0.602548	-0.561182	-0.671448	-0.521
C4A	0.346809	0.228368	-0.142766	-0.189491	-0.108
N5	-0.581155	-0.677167	-0.189397	-0.539849	-0.489
H5			0.304307	0.37613	0.344
C5A	0.519353	0.552962	0.156402	0.25892	0.124
C6	-0.429232	-0.498495	-0.453883	-0.511598	-0.235
H6	0.214339	0.19076	0.212203	0.187579	0.171
C7	0.195042	0.217973	0.221961	0.212641	0.051
C7M	-0.27782	-0.371534	-0.290236	-0.372657	-0.266
H71	0.09	0.09	0.09	0.09	0.09
H72	0.09	0.09	0.09	0.09	0.09
H73	0.09	0.09	0.09	0.09	0.09
C8	0.109434	0.120895	0.117511	0.099859	0.051
C8M	-0.261718	-0.377383	-0.280291	-0.374304	-0.275
H81	0.09	0.09	0.09	0.09	0.09
H82	0.09	0.09	0.09	0.09	0.09
H83	0.09	0.09	0.09	0.09	0.09
C9	-0.29736	-0.347211	-0.352379	-0.449271	-0.214
H9	0.185945	0.176699	0.194833	0.204074	0.175
C9A	-0.194394	-0.286893	0.046639	0.174393	0.095
N10	0.24429	0.356739	0.052737	-0.133253	-0.444
C10	0.112132	0.032016	0.349323	0.44234	0.49
C1'	-0.050017	-0.189131	-0.057074	-0.159231	-0.005
H1'	0.09	0.09	0.09	0.09	0.09
H1"	0.09	0.09	0.09	0.09	0.09
C2'	0.14	0.14	0.14	0.14	0.14
H2'	0.09	0.09	0.09	0.09	0.09
O2'	-0.65	-0.65	-0.65	-0.65	-0.65
HO2'	0.42	0.42	0.42	0.42	0.42
C3'	0.14	0.14	0.14	0.14	0.14
H3'	0.09	0.09	0.09	0.09	0.09
O3'	-0.65	-0.65	-0.65	-0.65	-0.65
HO3'	0.42	0.42	0.42	0.42	0.42
C4'	0.14	0.14	0.14	0.14	0.14

H4'	0.09	0.09	0.09	0.09	0.09
O4'	-0.65	-0.65	-0.65	-0.65	-0.65
HO4'	0.42	0.42	0.42	0.42	0.42
C5'	0.05	0.05	0.05	0.05	0.05
H5'	0.09	0.09	0.09	0.09	0.09
H5''	0.09	0.09	0.09	0.09	0.09
O5'	-0.65	-0.65	-0.65	-0.65	-0.65
HO5'	0.42	0.42	0.42	0.42	0.42

Supplementary Figures

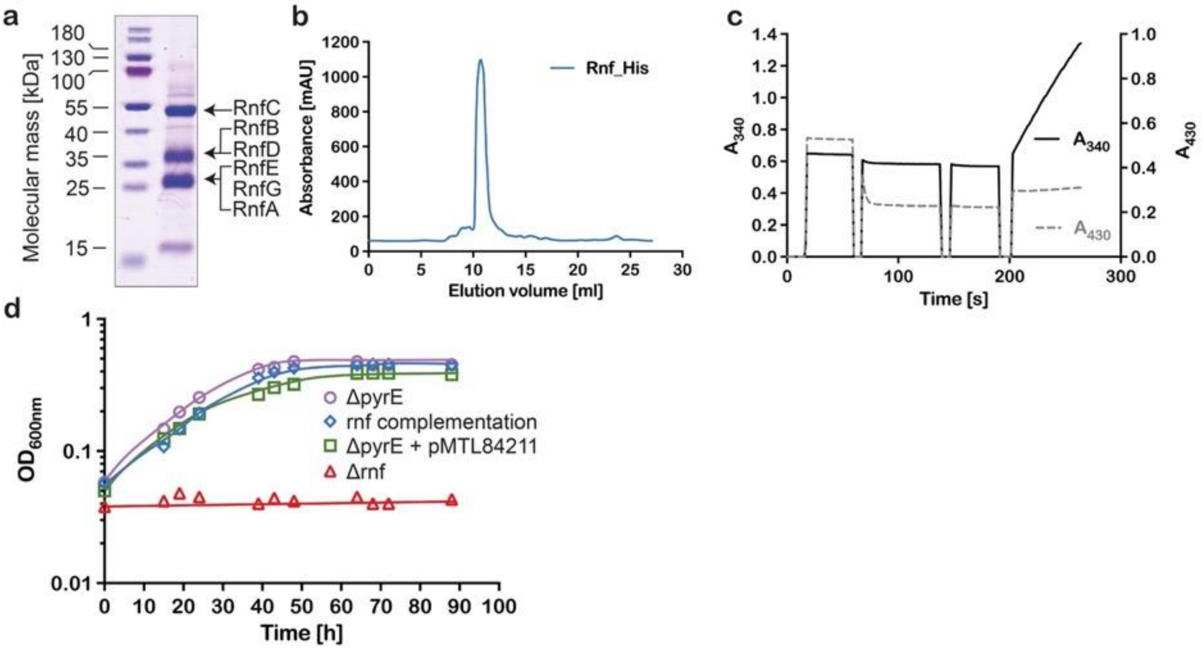

Supplementary Fig. 1 | Purification and characterisation of the Rnf complex from *A. woodii*. (a) 10 µg of Rnf complex containing a His-tag purified from *A. woodii* was separated in an SDS-PAGE. **(b)** Size exclusion chromatography profile of the purified Rnf complex from *A. woodii* on “Superdex 200 Increase™ 10/300”. **(c)** Measurement of Fd_{red}-dependent NAD^+ reduction catalysed by the Rnf complex from *A. woodii*. **(d)** Growth restoration of the *rnf* mutant on H_2 and CO_2 after complementation with the plasmid pMTL84211_Ppta_ack_Rnf-His. The *A. woodii* Δ*pyrE* mutant, the Δ*pyrE* mutant containing the vector pMTL84211 without the *rnf* operon, the Δ*rnf* mutant and the complemented strain Δ*rnf* were grown in complex medium under a H_2 + CO_2 [80:20 v/v] atmosphere with a pressure of 1.0×10^5 Pa.

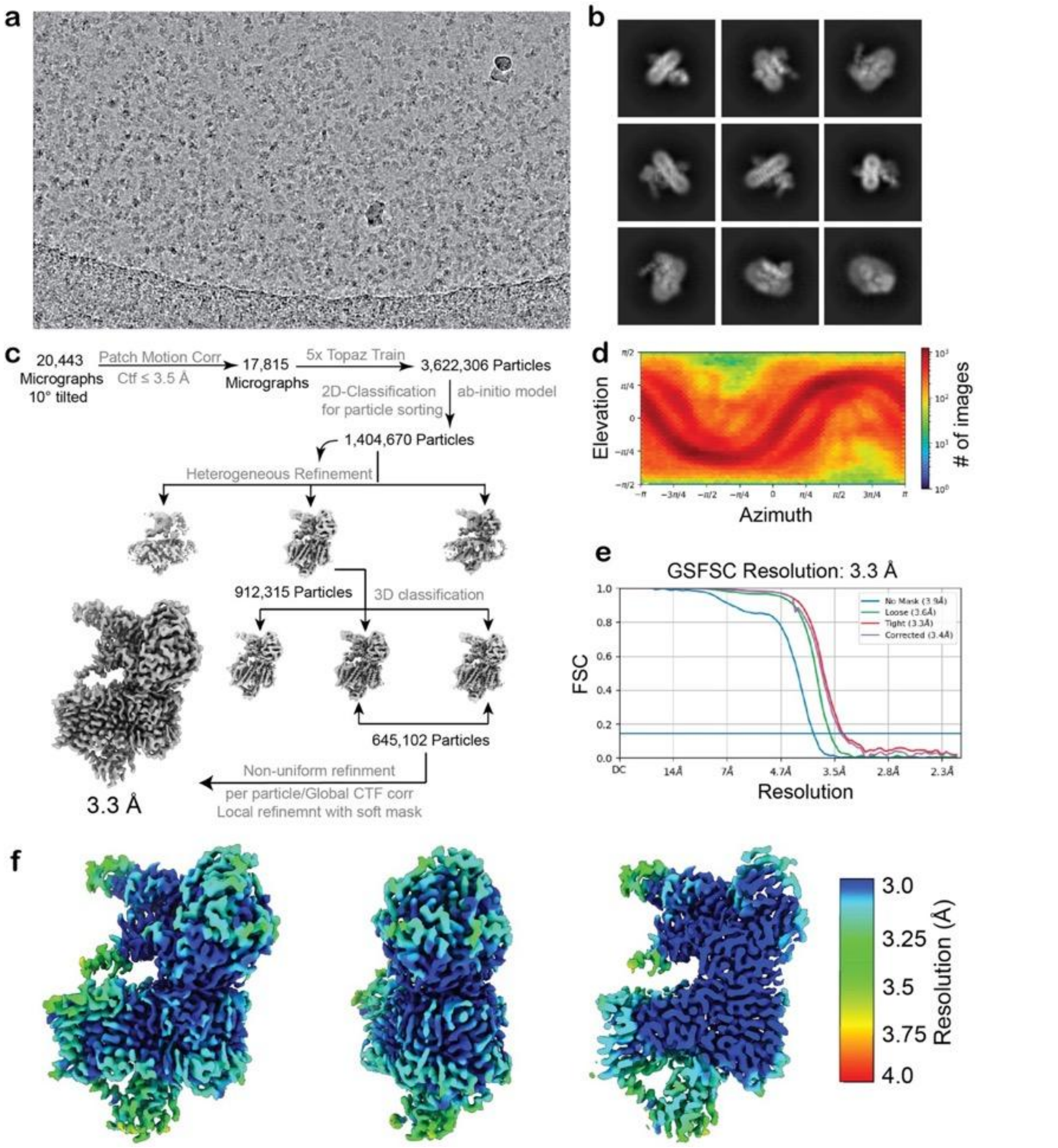

Supplementary Fig. 2 | Cryo-EM data collection and analysis of the Rnf complex with NADH bound. (a) A representative cryo-EM motion-corrected micrograph showing Rnf particles. **(b)** Reference-free 2D class averages revealing different views of the Rnf complex. **(c)** Overview of the cryo-EM data-processing scheme. A 10° tilted dataset was acquired to overcome the preferred orientation problem. **(d)** Angular distribution of the particles used for the final round of NU refinement. **(e)** Fourier shell correlation plot of final refined map with showing the global resolution (FSC = 0.143). **(f)** Local resolution as calculated by CryoSparc[25] mapped on the refined density (left, middle: front and side view, right: cut-open view of central section).

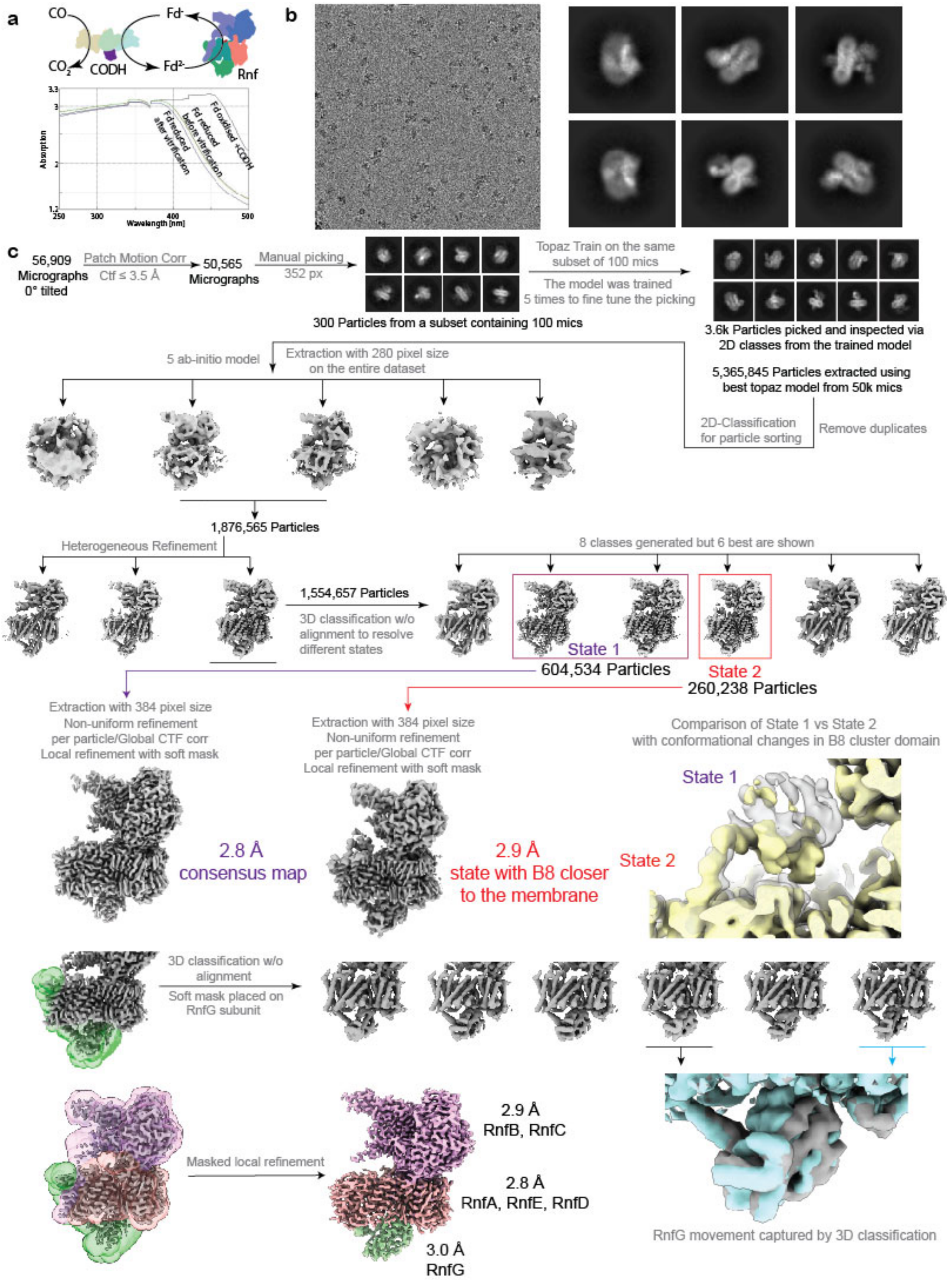

Supplementary Fig. 3 | Cryo-EM data collection and analysis of the Rnf complex reduced with pre-reduced Fd. (a) Cartoon scheme depicting the CODH enzyme extracting electrons from CO gas and transferring these electrons to oxidised Fd to produce reduced Fd. The Rnf is then incubated with reduced Fd to obtain a reduced protein complex. **(b)** A representative cryo-EM motion-corrected micrograph (4k x 4k) showing single Rnf complexes and reference-free 2D class averages revealing different views of the complex. **(c)** Overview of the cryo-EM data-processing scheme. Due to low number of particles on the grid, a large dataset was acquired to explore conformational changes. 3D classification revealed two conformations associated with the RnfB mobile domain containing the B8 cluster; one closer to the membrane (state 2) and one away (state 1) (see Methods for a detailed description of the processing pipeline).

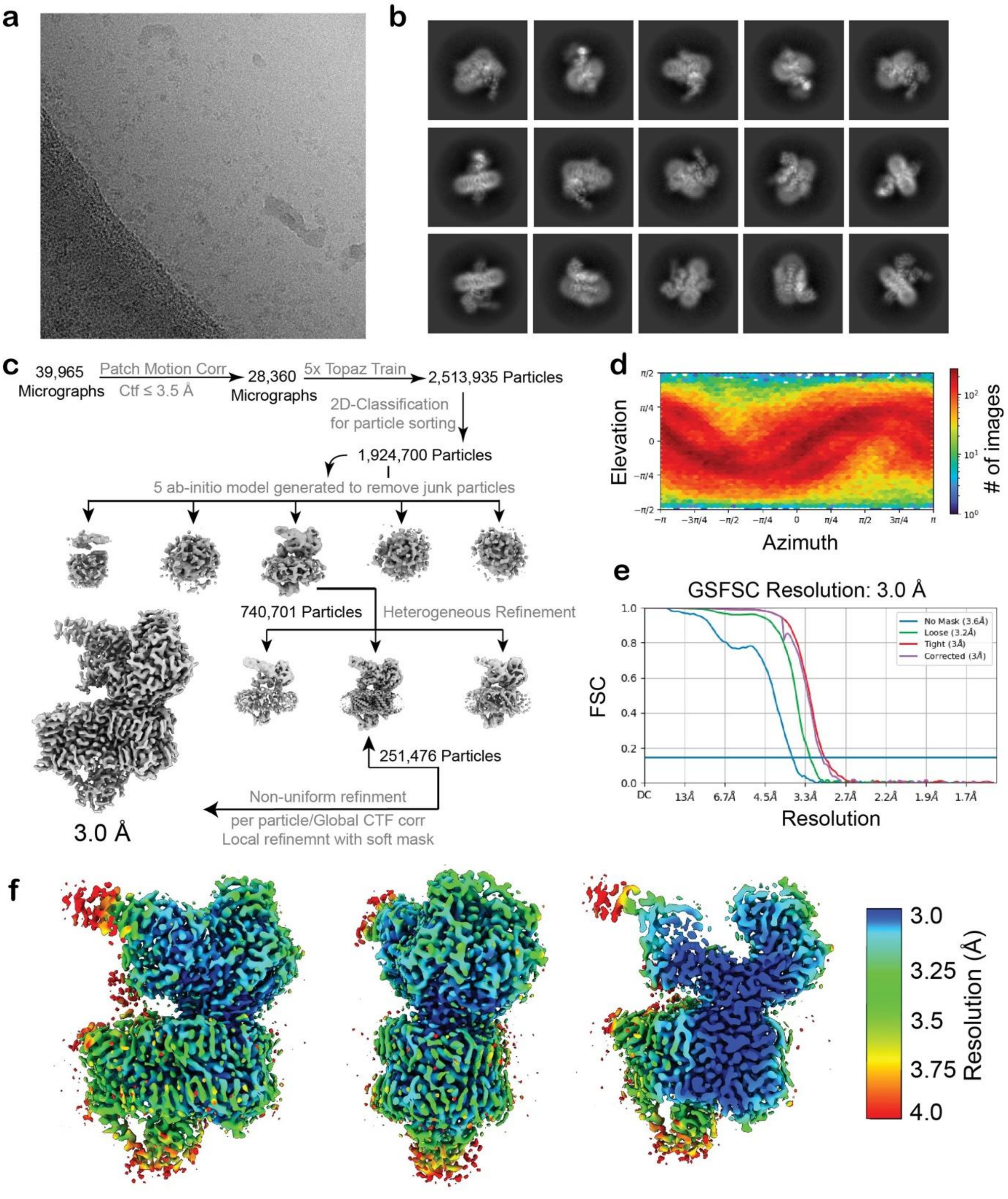

Supplementary Fig. 4 | Cryo-EM data collection and analysis of the *apo* state of the Rnf complex. (a) A representative cryo-EM micrograph showing Rnf particles. **(b)** Reference-free 2D class averages revealing different views of the Rnf. **(c)** Overview of the cryo-EM data-processing scheme. **(d)** Angular distribution of the particles used for the final round of NU refinement. **(e)** Fourier shell correlation plot of final refined map with showing the global resolution (FSC = 0.143). **(f)** Local resolution as calculated by CryoSparc[25] mapped on the refined density (*left*, middle: front and side view, *right*: cut-open view of central section).

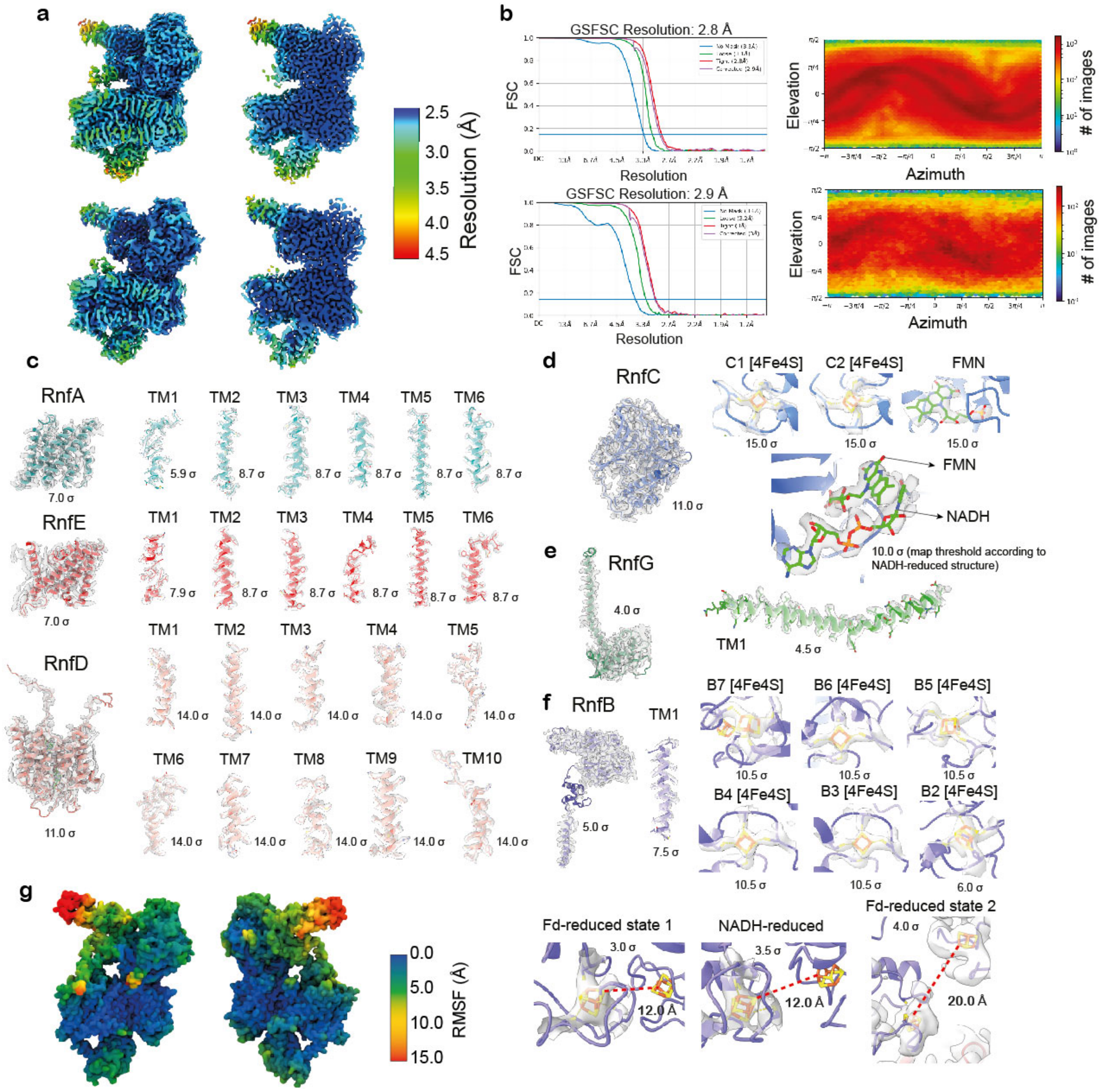

Supplementary Fig. 5 | Cryo-EM density and model quality. Representative regions of the Rnf subunits and their surrounding electron density maps are shown, along with map threshold in RMSD. **(a)** Local resolution as calculated by CryoSparc[25] mapped on the refined density (2.8 Å consensus map) of Rnf reduced with Fd_{red} taken from Fig. S3 (front and cut-open view of central section). **(b)** Fourier shell correlation and angular distribution plots of the Rnf complex structure (state 1 and state 2) reduced with pre-reduced Fd. **(c, d, e, f)** Representative regions of the Rnf subunits and their surrounding electron density maps are shown, indicating the corresponding contour level (σ) values. (**c**) The helices TM1 and 4 for RnfA/E subunit were found slightly disordered with moderate density fits, indicating their flexible nature. (**d**) The FMN-NADH bound state was only obtained for Rnf complex with bound NADH, whereas for the Rnf in the Fd reduced state and the *apo* state contain only FMN. (**f**) The position of the B8 cluster encased around the cryo-EM map is shown for the Fd-reduced (state 1 and 2) and NADH-reduced state of the Rnf complex. **(g)** Average root-mean-square fluctuations (RMSF) calculated from the MD simulations and mapped on the cryo-EM structure of the Rnf complex treated with reduced Fd.

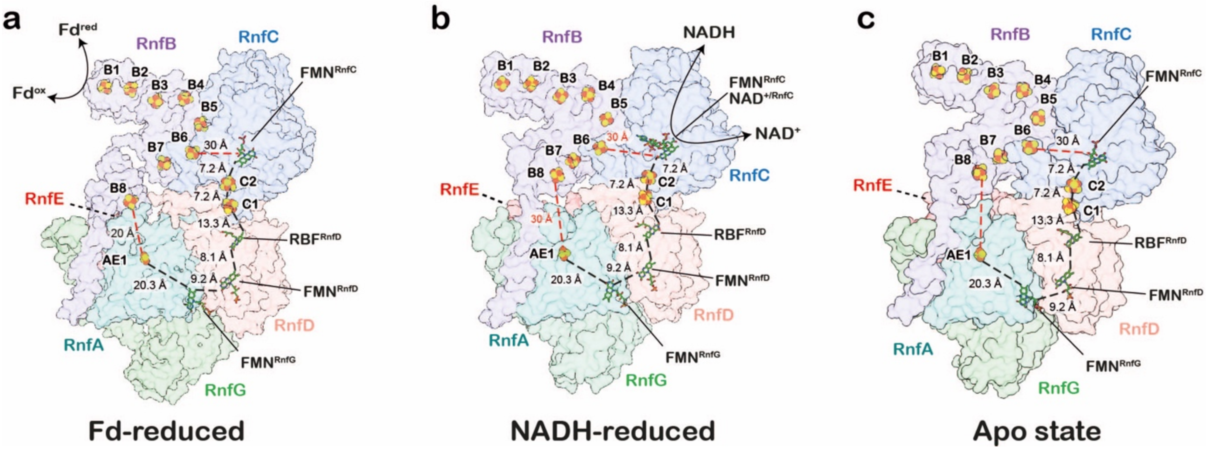

Supplementary Fig. 6 | Comparison of cofactor distances in the different states of the Rnf complex resolved by cryo-EM. (a) Rnf complex reduced with ferredoxin. **(b)** Rnf complex reduced with NADH. **(c)** Rnf complex in its *apo*-state. All structures are represented as transparent surfaces with *edge-to-edge* distances between the cofactors reported in ångströms.

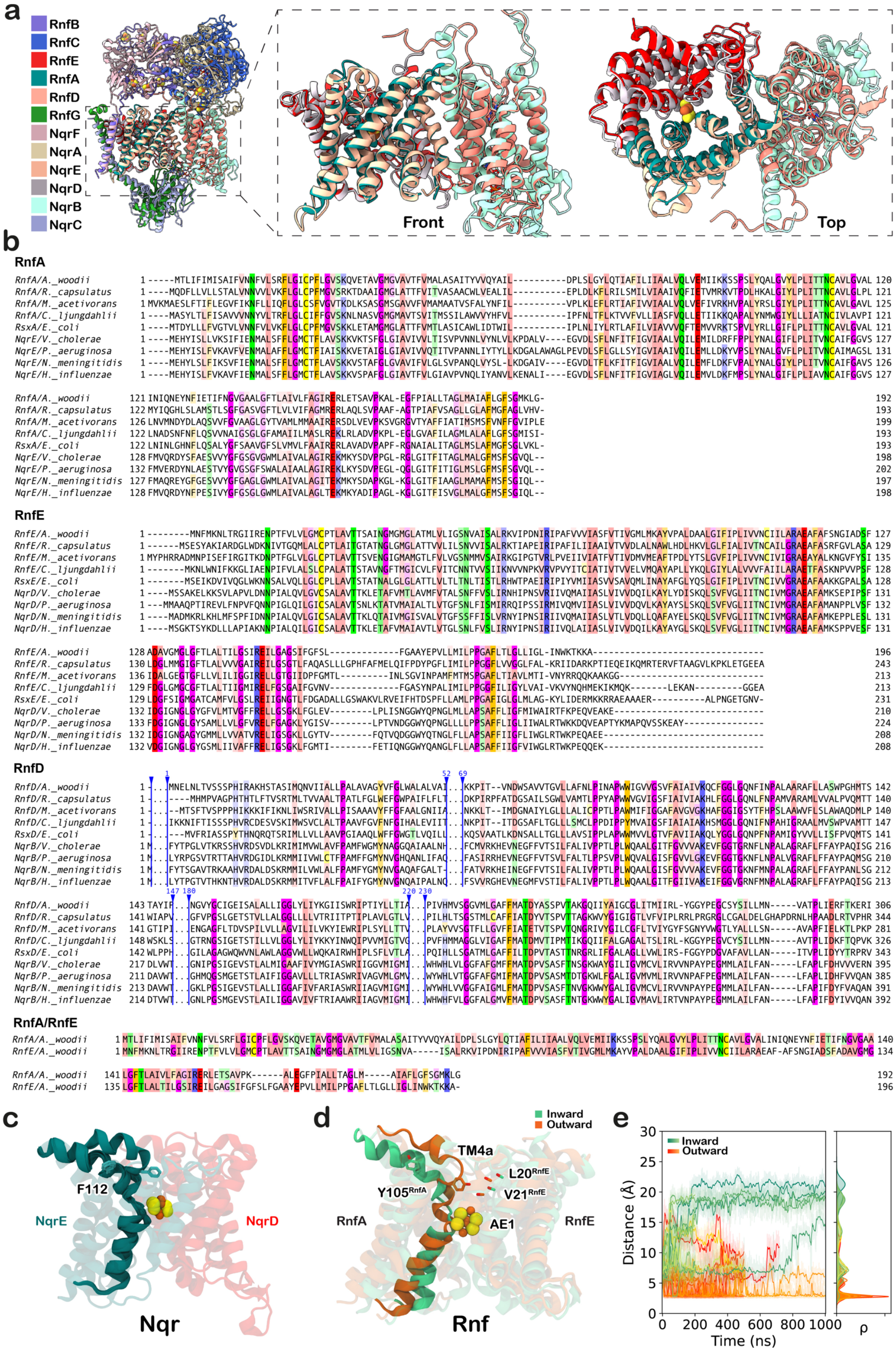

Supplementary Fig. 7 | Structural comparison of Rnf and Nqr and sequence conservation. (a) Superposition of the Rnf structure (PDB ID: 9ERI) with Nqr complex (PDB ID: 7XK3)[26]. The overall complex aligns with an RMSD of 4.75 Å, however, the membrane integral subunits, shown as closeup, are highly similar and align with an RMSD value of 1.18 Å. **(b)** The corresponding multiple-sequence alignment (MSA) of the membrane integral subunit of the Rnf complex (RnfA/E/D) and the Nqr complex (NqrB/D/E). Sequence identity between Rnf and Nqr: RnfA/NqrE = 37.0-48.4%; RnfE/NqrD = 29.1-39.8%; RnfD/NqrB = 21.8-31.4%. The RnfA/E, although being pseudo-symmetrical, show a sequence similarity of 29%. The MSA was performed with ClustalΩ[23]. Residues with up to 50% conservation are coloured. **(c, d, e)** Comparison of conformations from resolved cryo-EM structures of Nqr (PDB ID: 8A1T, 8A1U, 8A1V, 8A1W, 8A1X, 8A1Y, 8ACW, 8ACY)[27] (c) with MD inward/outward conformations (d), (e) Distance between the residues Y105^{RnfA}-V21^{RnfE} from MD simulations grouped either into inward (green) or outward (orange) conformations.

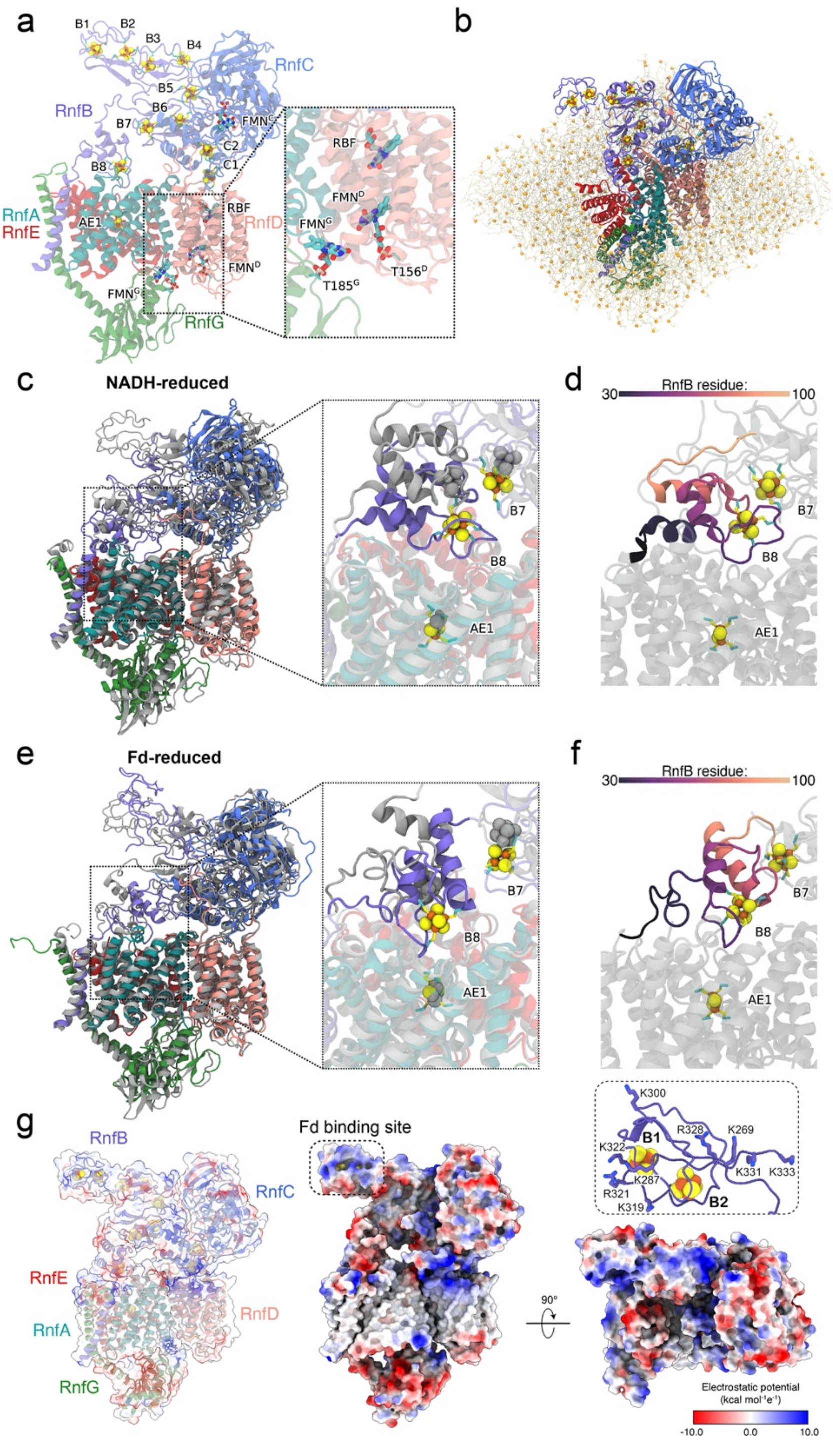

Supplementary Fig. 8 | MD simulations of the Rnf complex. (a) Structural overview of the Rnf system used for MD simulations. Subunits RnfA (teal), RnfB (purple), RnfC (blue), RnfD (pink), RnfE (red), RnfG (green) are shown in cartoon representation. The iron-sulphur clusters are represented as spheres (sulphur:yellow, iron:orange), while the cofactors FMN and RBF are shown as sticks. *Inset*: The FMN^{G} and FMN^{D} were covalently linked to $T185^{RnfG}$ and $T156^{RnfD}$, respectively. **(b)** The protein system embedded in a POPC membrane. Waters and ions are omitted for clarity. **(c,e)** Comparison of structures from cryo-EM experiment (grey) and equilibrated MD simulations. The MD simulations were started either from **(c)** the NADH- or **(e)** the Fd-reduced cryo-EM structures. *Inset:* close-up of B8 cluster binding region of RnfB (purple) showing the shift in position from MD simulations as compared to initial cryo-EM structure (grey). **(d, f)** Secondary structure of RnfB region binding the B8 cluster from MD simulations started from **(d)** the NADH- or **(f)** the Fd-reduced structures. Residue range of 30 (dark purple) to 100 (yellow) is shown. **(g)** Electrostatic potential on the Rnf surface shows a positive electrostatic potential region that could stabilise ferredoxin interaction. *Inset:* The C-terminal domain of RnfB contains several positive Lys/Arg residues.

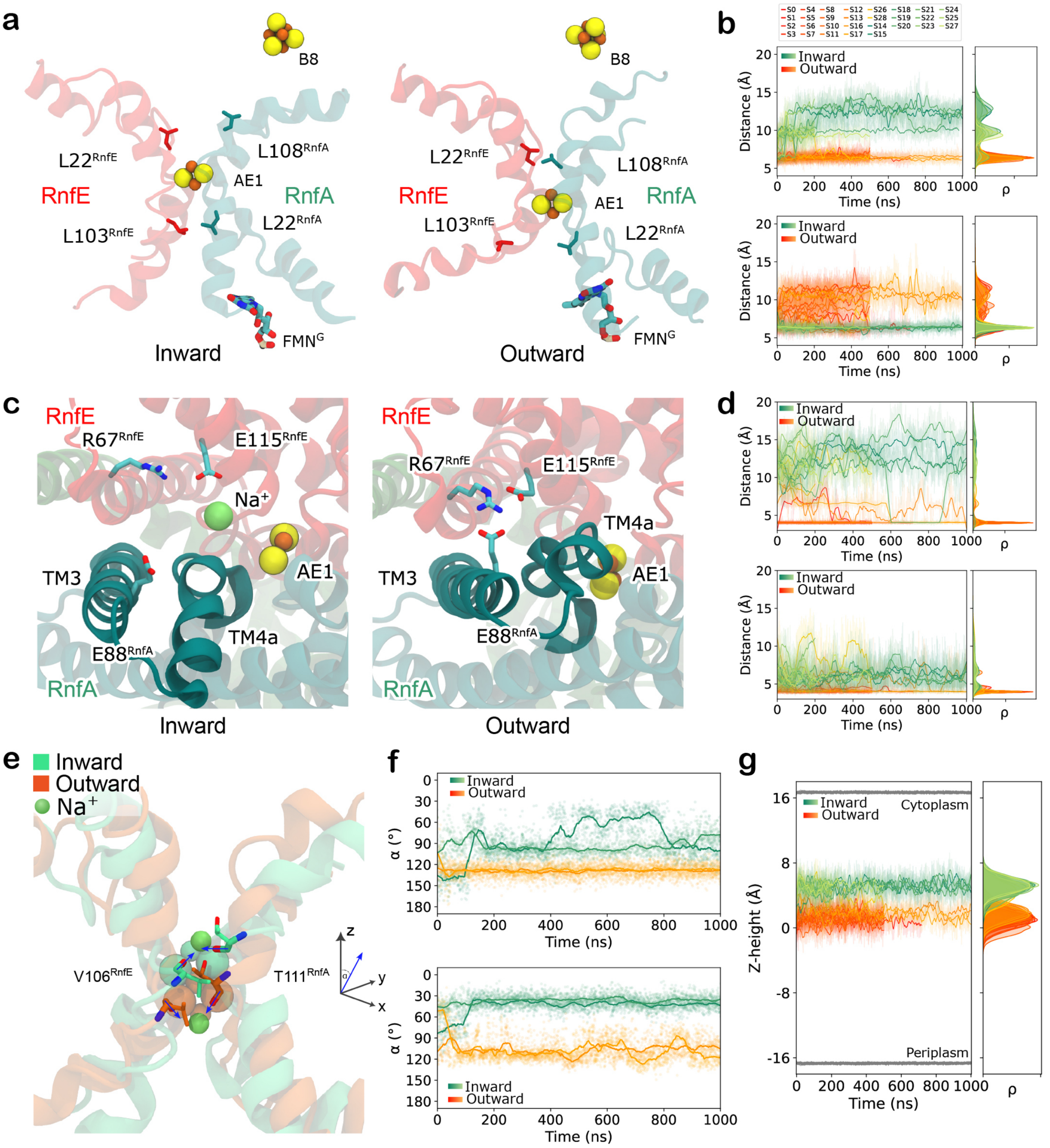

Supplementary Fig. 9 | Characterisation of the inward and outward conformations. (a) Inward (*left*) and outward (*right*) conformations of TM4 and TM1 helices from RnfA (teal) and RnfE (red). Conserved hydrophobic residues ($L22^{RnfA}$, $L22^{RnfE}$, $L103^{RnfE}$, $L108^{RnfA}$) can adopt different conformations depending on the helix conformation, controlling access to the AE1 cluster. **(b)** Distance between the gating hydrophobic residues *top:* $L22^{RnfE}$ and $L108^{RnfA}$; and *bottom*: $L22^{RnfA}$ and $V106^{RnfE}$. **(c)** Snapshots from the inward (*left*) and outward (*right*) conformations showing different ion pair conformations for $R67^{RnfE}$, $E88^{RnfA}$, and $E115^{RnfE}$. **(d)** Distances between the $R67^{RnfE}$-$E88^{RnfA}$ (*top*) and $R67^{RnfE}$-$E115^{RnfE}$ (*bottom*) ion pairs. **(e)** Sodium binding in the buried binding site is stabilised by backbone carbonyls $V106^{RnfE}$ and $T111^{RnfA}$ in the inward (green) and outward (orange) conformations. **(f)** Angle between the backbone carbonyls from $V106^{RnfE}$ (*top*) and $T111^{RnfA}$ (*bottom*), and the Z-axis (in °) from MD simulations with a reduced AE1 cluster (S13-S16, see Supplementary Table 6). **(g)** The Z-coordinate (in Å) of the AE1 cluster relative to the membrane. The average Z-coordinate (in Å) of the upper and lower membrane leaflets are shown in grey.

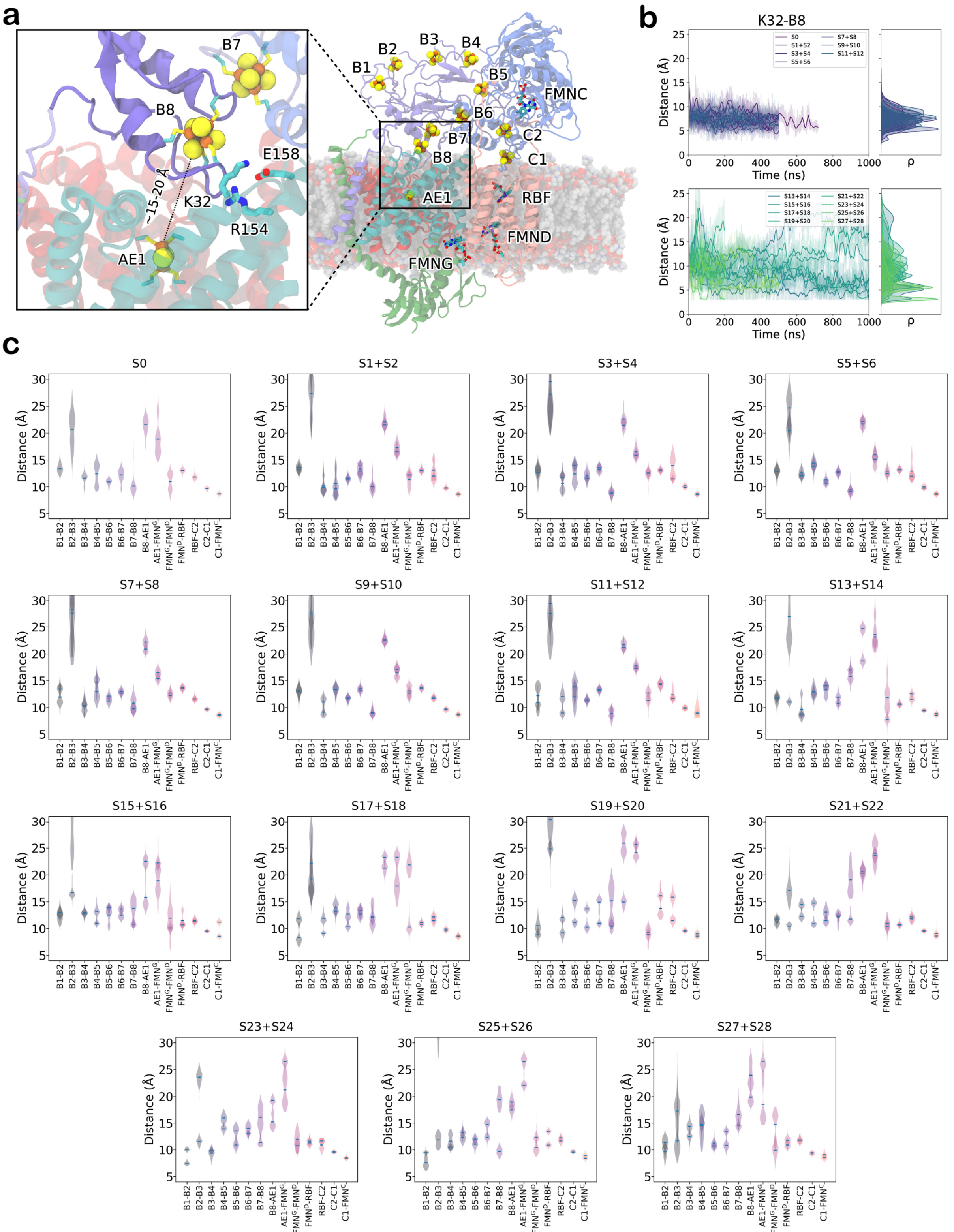

Supplementary Fig. 10 | Summary of cofactor distances from MD simulations. (a) Structural overview of the Rnf showing the location of the cofactors after MD equilibration (S15). *Inset:* A snapshot from MD simulation S15 showing the flexible domain of RnfB, containing the B8 cluster, binding close to the membrane subunits of RnfA and RnfE. Two positively charged residues ($K32^{RnfA}$, $R154^{RnfA}$) are involved in electrostatic interaction with the iron-sulphur cluster. **(b)** Distances between $K32^{RnfA}$ and B8-cluster of MD simulations starting from NADH- (*top*) or Fd-reduced (*bottom*) structures. See Supplementary Table 6 for the simulation details. **(c)** Violin plots showing all distances between the cofactors.

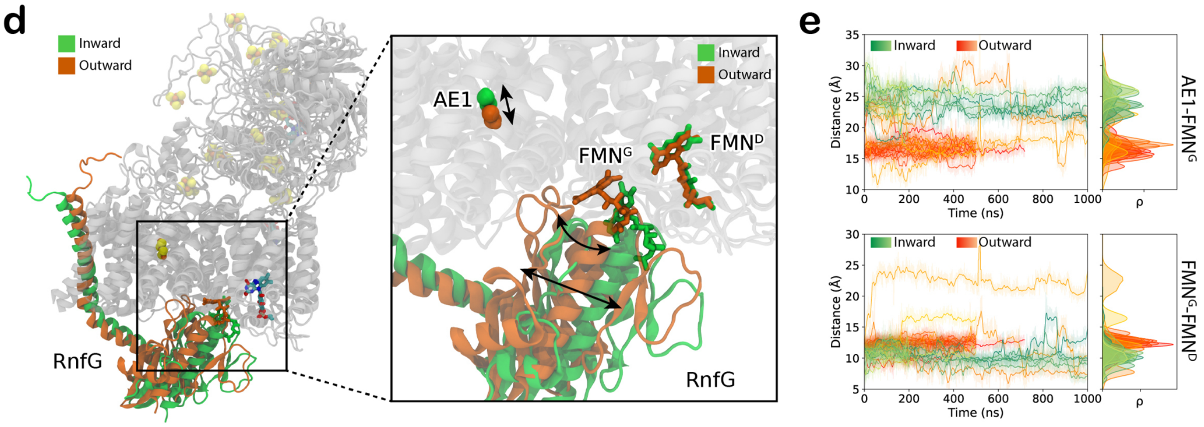

Supplementary Fig. 10 | Summary of cofactor distances from MD simulations (continued). (d) Snapshot of RnfG from MD simulations with RnfA/E either in inward (green) or in outward (orange) conformation. *Inset:* closeup view of the cofactor FMN^G showing two distinct conformations. In outward-conformation it moves closer to the AE1-center, whereas in inward-conformation it moves closer to FMN^D. **(e)** *Edge-to-edge* distance analysis shown for AE1-FMN^G (*top*) and FMN^G-FMN^D (*bottom*) from all MD simulations grouped either into inward (green hues) or outward (orange hues) conformation.

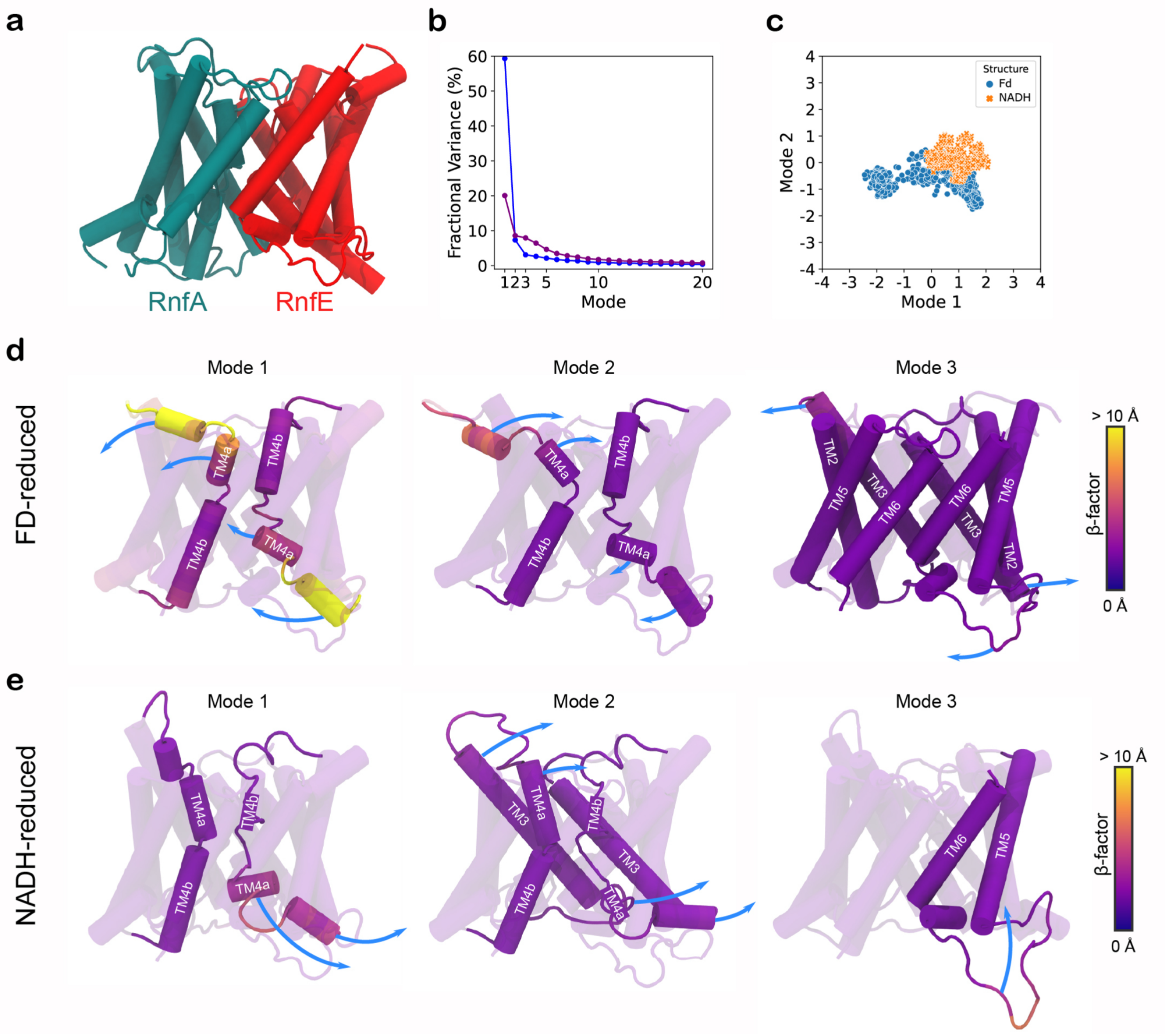

Supplementary Fig. 11 | Global dynamics of the Rnf complex from principal component analysis (PCA) of the MD simulations. (a) Overview of subunits used for the PC analysis. RnfA is shown on the left (in teal) and RnfB to the right (in red). **(b)** Scree plot showing the fractional variance of the obtained normal modes. **(c)** Projection of the MD simulations onto the principal components (PC) - modes 1 and 2. **(d)** The main PC modes of MD simulations based on the Fd-reduced structure with the largest movements of helices TM1 and TM4, showing symmetric (mode 2) or asymmetric (mode 1) motion. Rotational motion of the outer helices (TM2, TM3, TM5) (mode 3). **(e)** The main PC modes from MD simulations of the NADH-reduced structure show overall similar dynamics as in the Fd-reduced simulations, but lower β-factors, indicating a smaller movement. See Supplementary Movies 3 and 4 for the conformational switching and PCA.

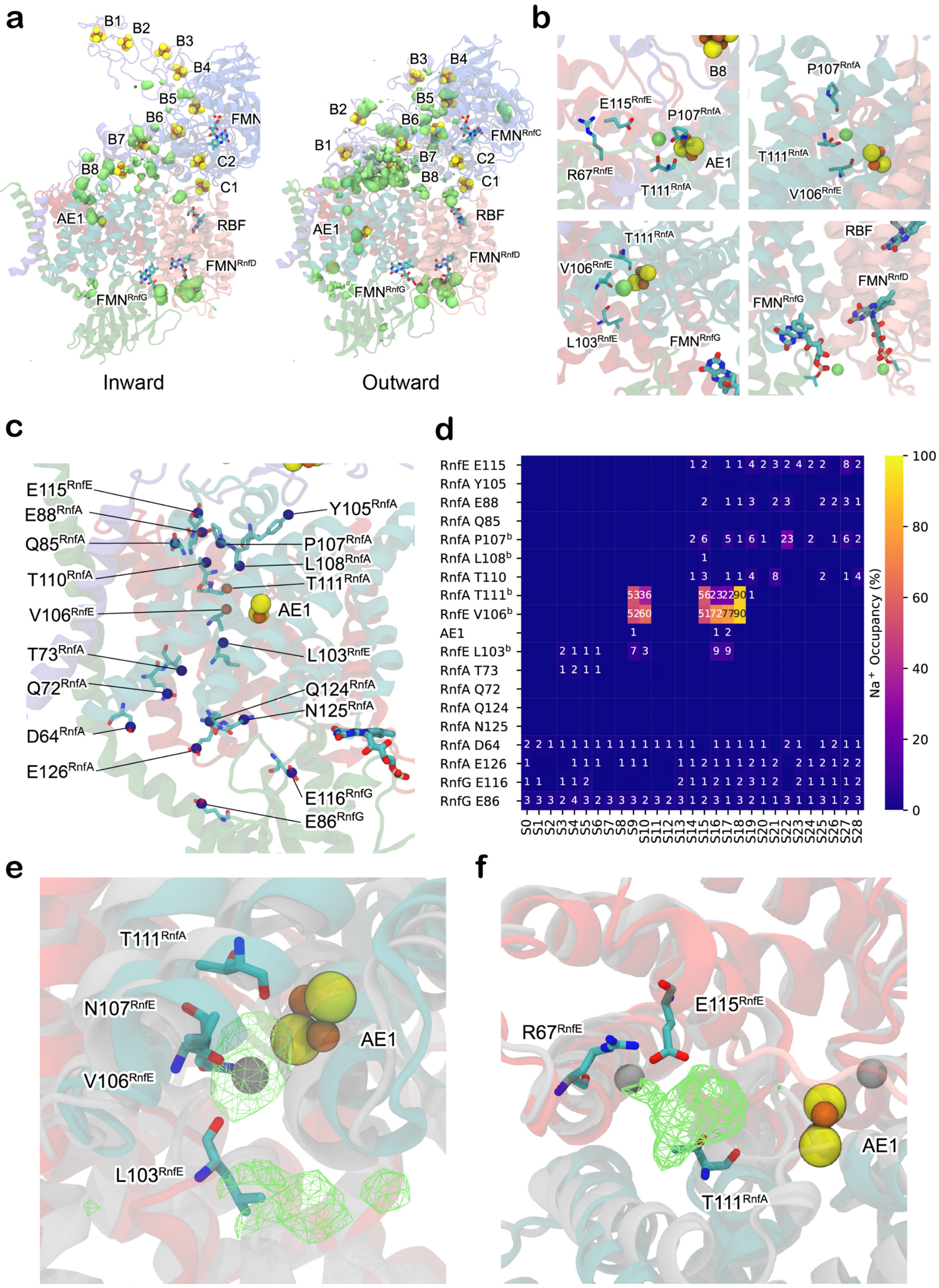

Supplementary Fig. 12 | Sodium binding in the membrane domain of Rnf. (a) Analysis of the Na$^+$ occupancy from combined MD simulations of the inward (*left*) and outward (*right*) conformations. The average Na$^+$ occupancies were calculated using the VolMap tool in VMD[8] shown as green-coloured densities. **(b)** Snapshots from MD simulations showing key residues interacting with the Na$^+$ ion. *Top, left*: the cytosolic side, with Na$^+$ ion bound between E115^{RnfE} and T111^{RnfA}. *Top*, *right*: buried binding site in the inward conformation, with the Na$^+$ ion bound to the carbonyl backbone of T111^{RnfA} next to the AE1 cluster. *Bottom*, *left*: the outward conformation, with Na$^+$ ion bound next to the carbonyl backbone of V106^{RnfE} and L103^{RnfE}. *Bottom, right:* Na$^+$ ion next to the phosphate groups of FMN^G and FMN^D on the extracellular side. **(c)** Subunits RnfA/E with potential sodium ion interactions marked in purple spheres. **(d)** Na$^+$ occupancy during MD simulations with the residues shown in panel **c** (see Supplementary Table 6). The occupancies were calculated based on the fraction of frames with a sodium ion within 3 Å of a particular residue (*b* - for only backbone interaction). **(e, f)** Superposition of Na$^+$ binding sites from cumulative MD simulations snapshots (green densities, obtained using the VMD VolMap tool for 1 frame/ns of the MD trajectory, from simulations S17/S18) and cryo-EM structures (grey spheres). Panel **(e)** shows the periplasmic side of the RnfA/E subunit, and panel **(f)** shows the cytoplasmic side.

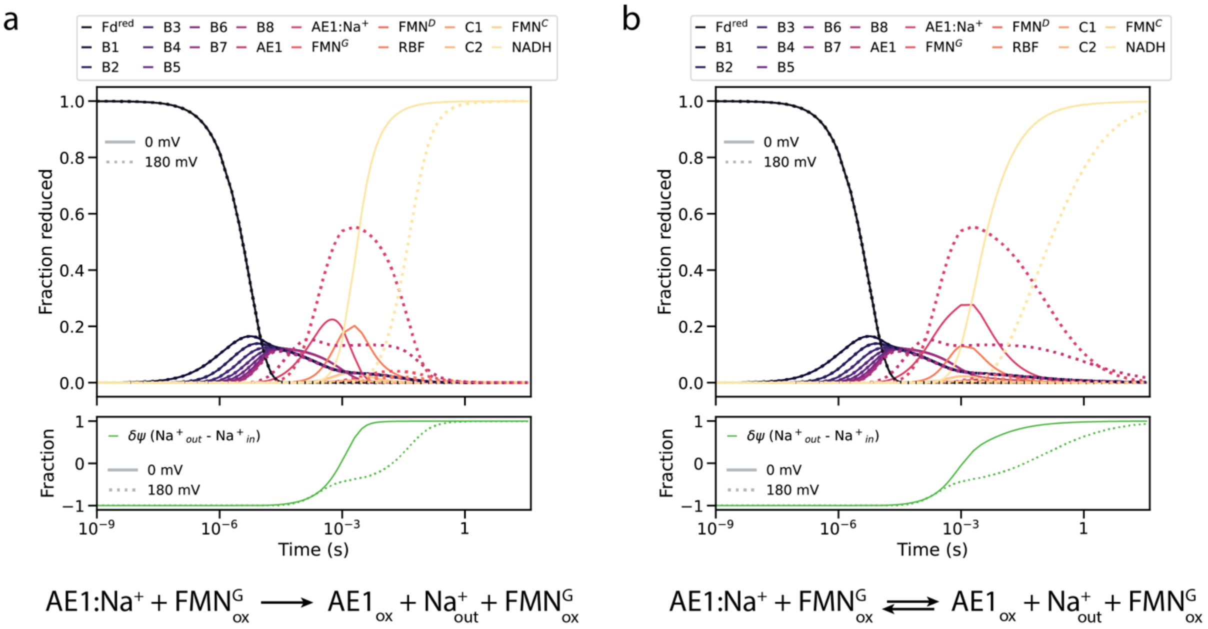

Supplementary Fig. 13 | Kinetic simulations of the sodium translocation process with **(a)** an irreversible, and **(b)** a reversible Na^+ release steps.

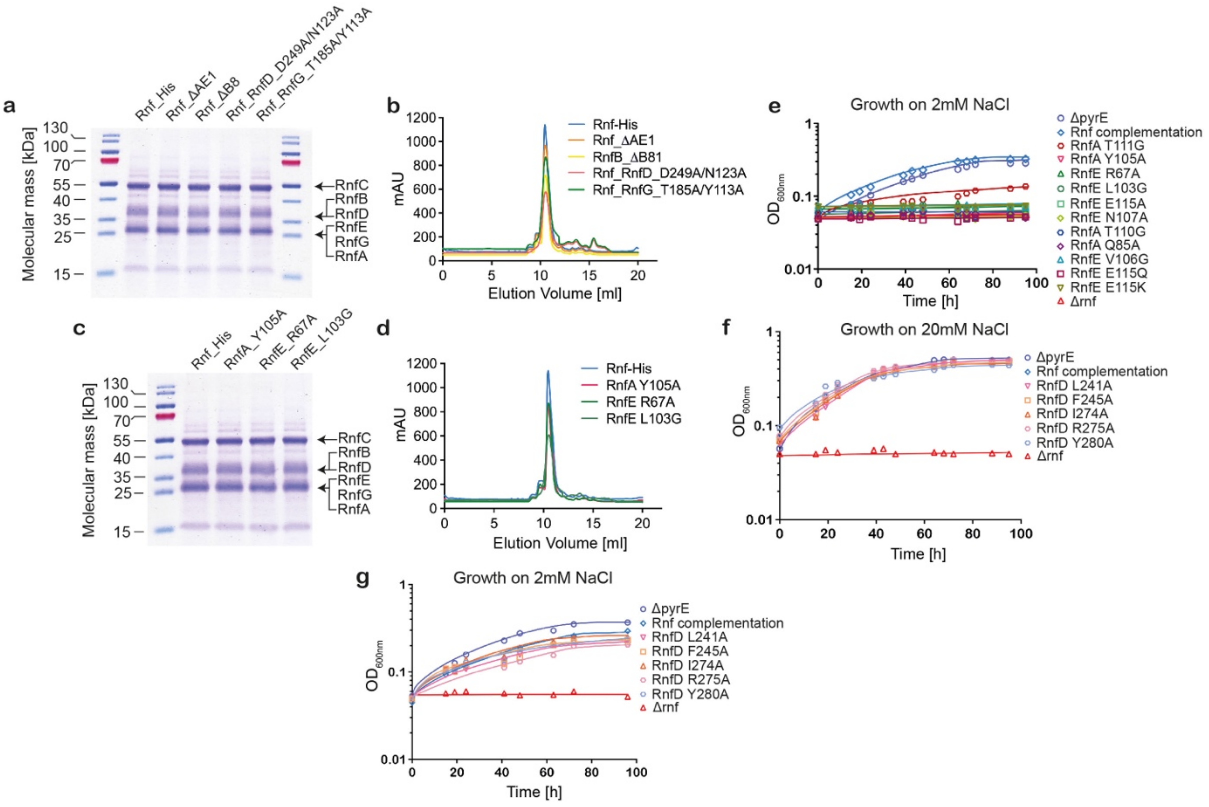

Supplementary Fig. 14 | Purification and characterisation of Rnf variants from *A. woodii*. (a, b) 10 µg of Rnf electron transfer pathway variants containing a His-tag purified from *A. woodii* was separated in an SDS-PAGE. Size exclusion chromatography profile of the purified variant Rnf complexes from *A. woodii* on "Superdex 200 Increase™ 10/300". **(c, d)** 10 µg of Rnf variants with alterations in the proposed Na^+ binding site containing a His-tag purified from *A. woodii* was separated in an SDS-PAGE. Size exclusion chromatography profile of the purified mutant Rnf complex from *A. woodii* on "Superdex 200 Increase™ 10/300". **(e)** Rnf strain containing mutations of residue involved in Na^+ binding and translocation (in RnfA/E subunits) did not grow on H_2 and CO_2 at 2 mM NaCl **(f, g)** Rnf strain containing mutations of residue in RnfD subunits grew as wild type when grown on 20 mM and 2 mM NaCl with H_2 and CO_2.

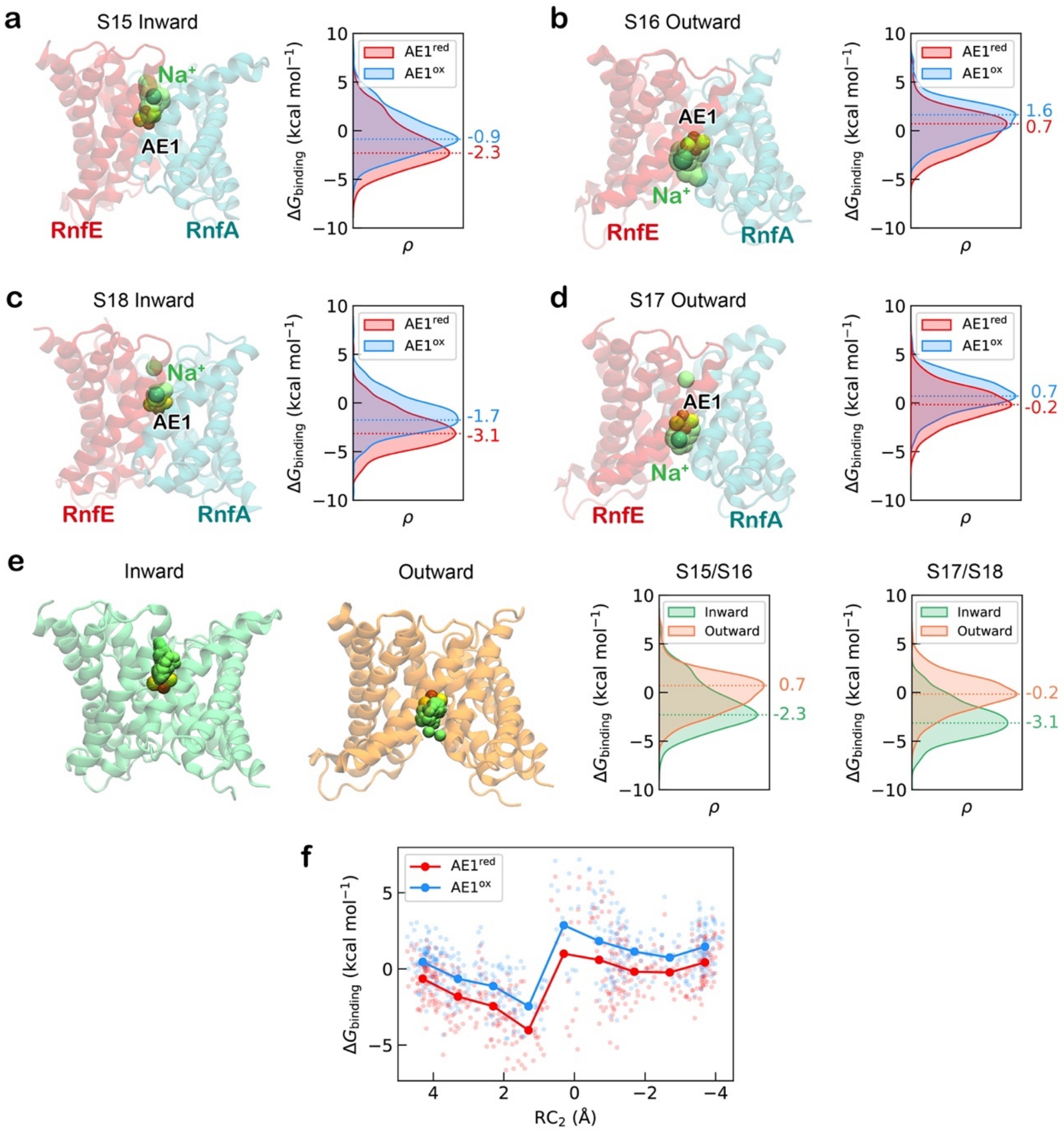

Supplementary Fig. 15 | Sodium binding to the RnfA/E dimer. (a,b,c,d) The data shows the binding affinity of Na^+ ions in different redox states of the AE1 cluster (red: reduced; blue: oxidised), and in different alternate-access conformations of the RnfA/E dimer. Only snapshots with Na^+ bound were considered. The clustered snapshots were generated from MD simulations performed with the reduced AE1 cluster (simulations S15-S18, see Supplementary Table 6), whereas the effect of the AE1 oxidation was modelled by switching the AE1 charges to the corresponding oxidised state, as no spontaneous Na^+ binding was observed in MD simulations with an oxidised AE1 cluster. **(e)** Simulations grouped by inward (green) and outward (orange) conformations, showing the data with reduced AE1 cluster. **(f)** Sodium binding free energy (from PBSA/MM) along the sodium pathway across the RnfA/E subunits, sampled during the free energy calculations of the inward/outward transition (see Supplementary Figs. 18-20), with different redox states of the AE1 cluster (red: reduced; blue: oxidised). The solid lines/points represent the block average along the reaction coordinate, with a bin width of 1 Å (RC_2, defined as the distance between sodium and the centre of mass of RnfA/E, projected onto the Z-axis, with $RC_2 > 0$ being the intracellular side, and $RC_2 < 0$ the extracellular side, see also Supplementary Fig. 18).

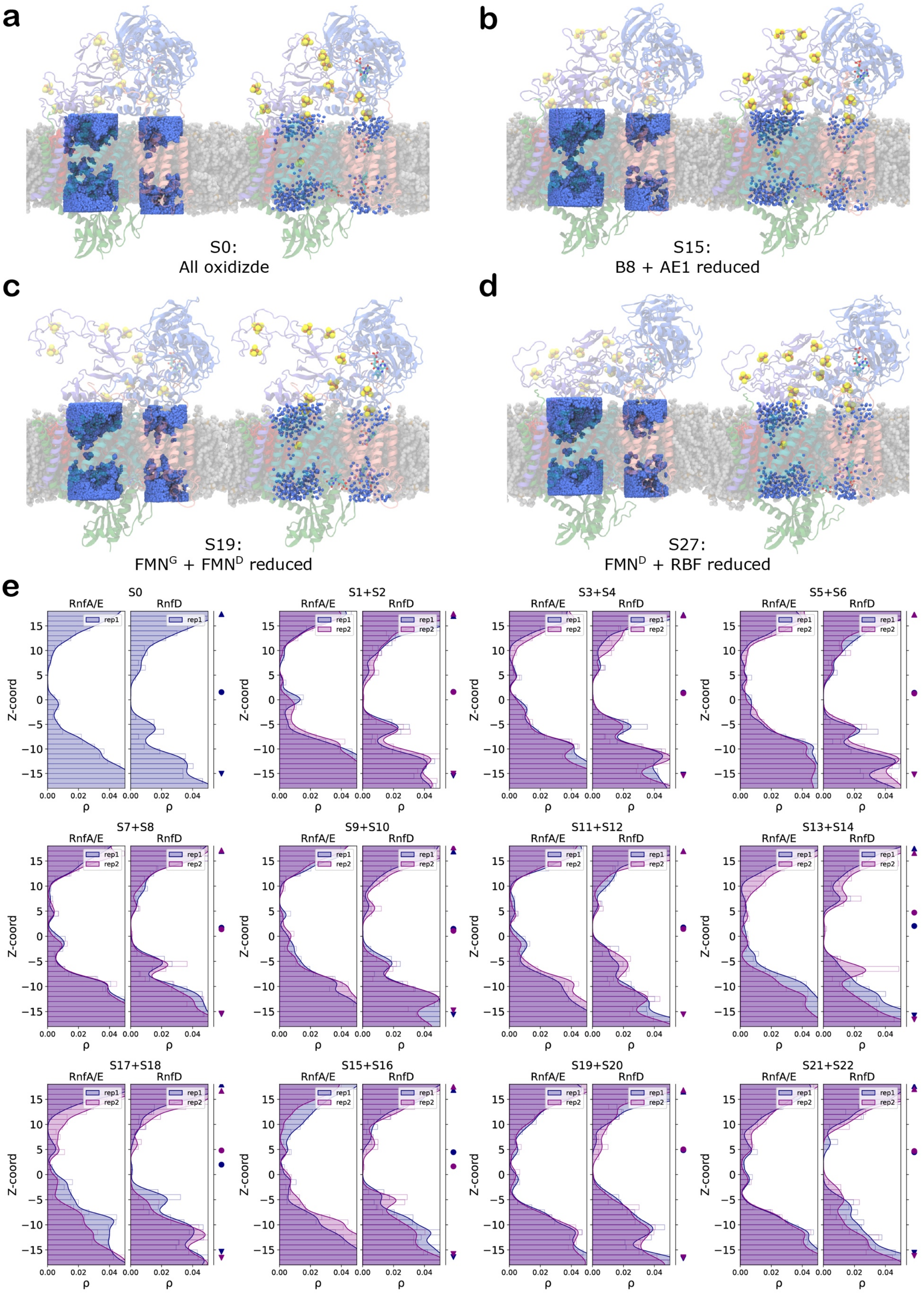

Supplementary Fig. 16 | Overview of hydration in the membrane subunits of Rnf. (a,b,c,d) *Left:* Hydration averaged over the MD ensemble, showing water molecules in the subunits RnfA, RnfE, and RnfD. The final 100 ns of each MD simulation with 1 ns/frame are shown. *Right:* The hydration of a final snapshot from the MD simulations. The water selection was defined by a cylinder cantered either to the subunits RnfA/E or RnfD, with the radius of the shape set to r = 14 Å or r = 11 Å, respectively. **(e)** Density of water molecules in RnfA/E (*left*) and RnfD (*right*) projected onto the Z-coordinate (in Å, perpendicular to the membrane plane). The mean position of the upper and lower membrane leaflet, and the AE1 cluster are indicated by triangles and circles, respectively.

Supplementary Fig. 16 | Overview of hydration in the membrane subunits of Rnf. (continued).

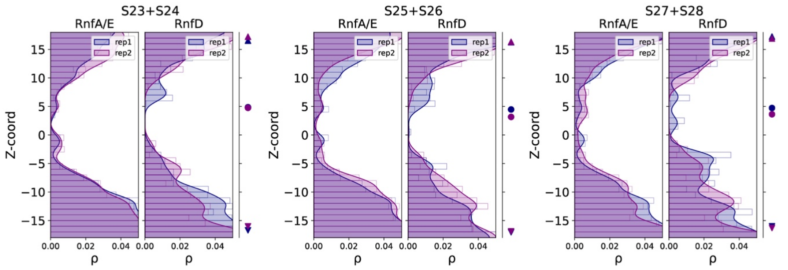

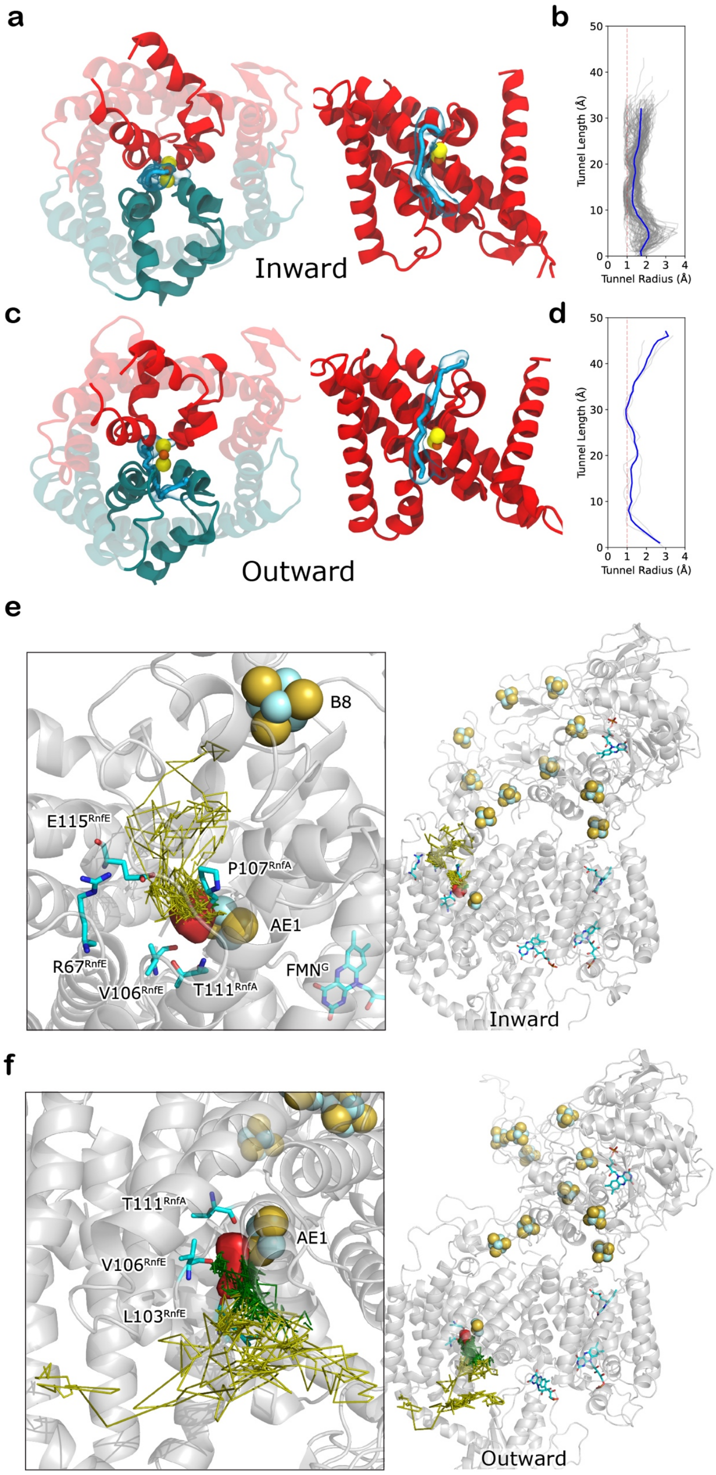

Supplementary Fig. 17 | Ion channel analysis in RnfA/E. (a, c) Ion translocation pathways, characterised using tunnel analysis in CAVER[16], showing a continuous pathway from (**a**) the inward and **(c)** outward sides of RnfA/E. **(b,d)** The tunnel radius of the individual pathways (in grey) and the average tunnel radius (blue) in the **(b)** inward and (**d**) outward conformations. The analysis was performed on MD simulations S15 and S16 (see Supplementary Table 6). **(e,f)** Analysis of Na^+ ion trajectories during MD simulations S15 **(e)** and S16 **(f)**. The pathways followed by the ion are shown as yellow and green lines, while the Na^+ cluster detected by AQUA-DUCT[17], is shown as a red surface.

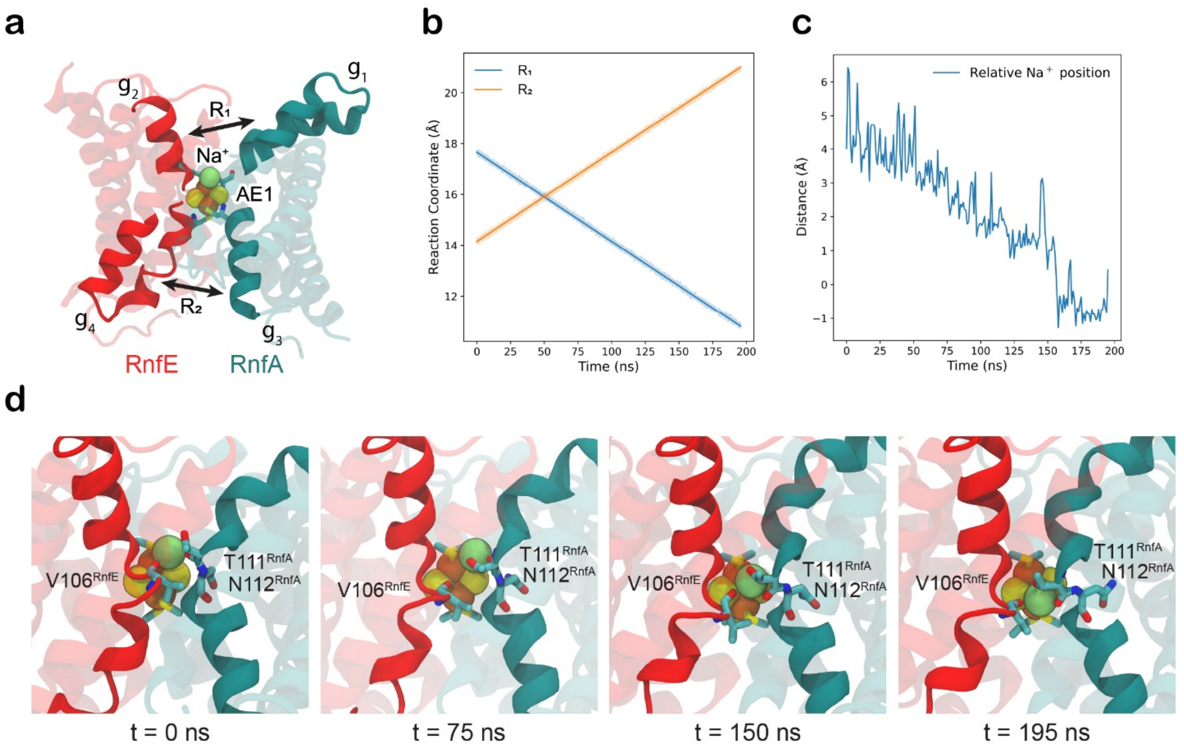

Supplementary Fig. 18 | Steered molecular dynamics exploration of the inward/outward transition. (a) Overview of the reaction coordinates (R_1 and R_2) used for exploring the inward to outward transition of the RnfA/E subunits by steered molecular dynamics (SMD) simulations. R_1 was defined as the Cα mean distance between helices g_1 (residues Q85^{RnfA} to T110^{RnfA}) and g_2 (residues C108^{RnfE} to A118^{RnfE}), while R_2 was defined as the Cα mean distance between helices g_3 (residues T80^{RnfE} to V105^{RnfE}) and g_4 (residues A116^{RnfA} to S126^{RnfA}). See Supplementary Methods for details. **(b)** The reaction coordinate R_1 (blue) and R_2 (orange) during the SMD simulations. **(c)** Distance of the Na^+ ion to the centre of the RnfA/E subunits, projected on the Z-axis shows how the ion moves from the intracellular side to the extracellular side during the SMD simulation. **(d)** Snapshots from the SMD simulation, showing the Na^+ ion and coordinating residues at different timepoints.

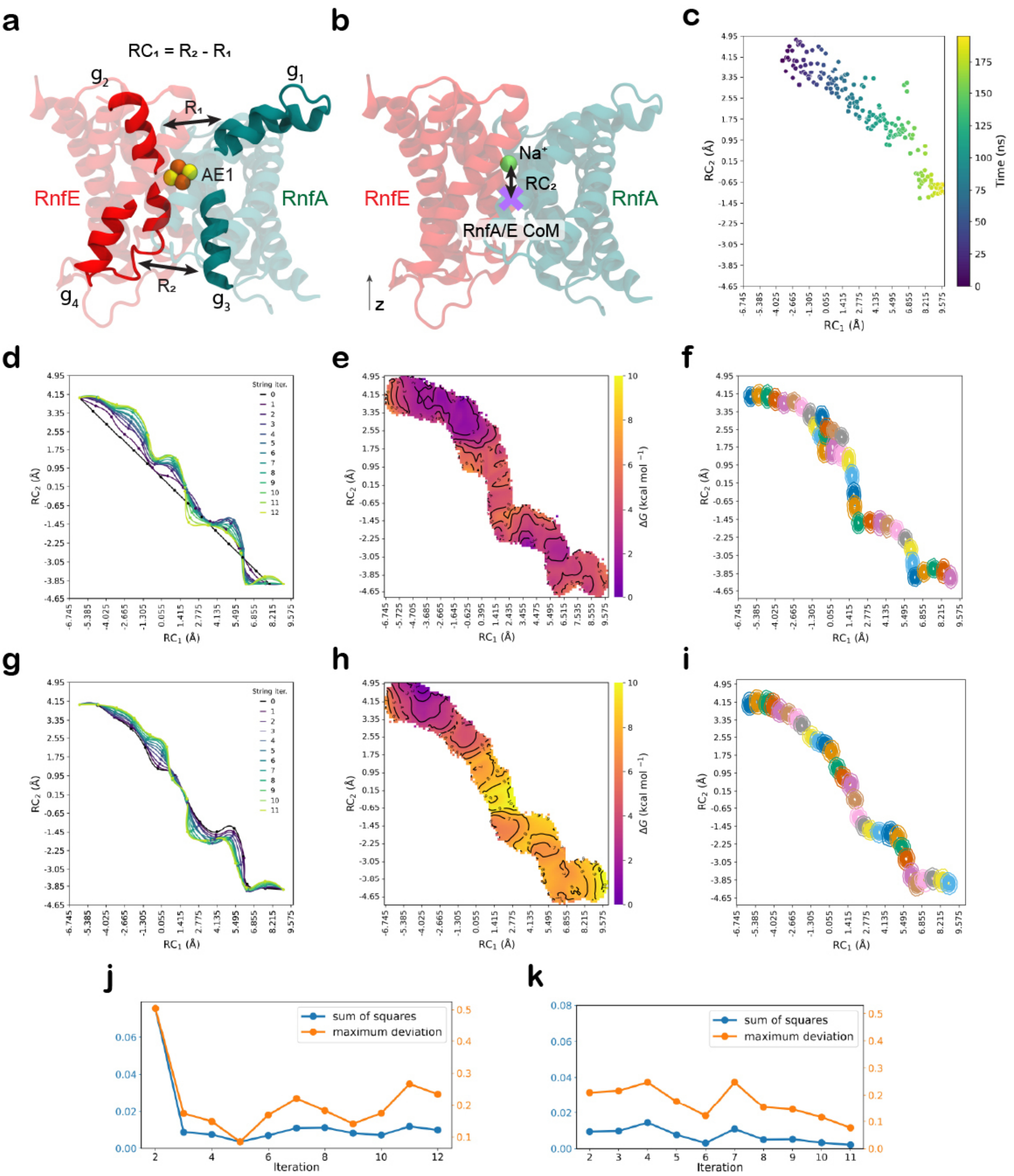

Supplementary Fig. 19 | Free energy calculations of the inward-outward transition. (a, b) 2D reaction coordinates (RC) used for the string simulations. **(a)** RC_1 is defined as the linear combination of two distances R_2-R_1. R_1 is the mean distance between Cα atoms of helices g1 (residues Q85^{RnfA}-T110^{RnfA}) and g2 (residues C108^{RnfE}-A118^{RnfE}), while R_2 is the mean distance Cα atoms of helices g3 (residues T80^{RnfE}-V105^{RnfE}) and g4 (residues L116^{RnfA}-E126^{RnfA}). **(b)** RC_2 is defined as the distance between the Na^+ ion and centre of mass (CoM) of RnfA/E Cα atoms projected onto the Z-axis. **(c)** Projection of the SMD trajectory onto the 2D reaction coordinates RC_1 and RC_2 (see Supplementary Fig. 18 and Supplementary Methods). Initial structures for the string simulation windows were chosen from the SMD simulation by equidistant spacing in the RC_1 dimension. **(d, g)** Optimisation of the string pathway with a **(d)** reduced or **(g)** oxidised AE1-cluster. The initial pathway (iteration 0) of the oxidised state was started from the fifth string iteration of the reduced state. **(e, h)** 2D Free energy landscape of the **(e)** reduced and **(g)** oxidised state simulations. **(f, i)** Phase space sampling of the converged string in **(f)** the reduced and **(i)** oxidised states. **(j, k)** Sum of squares (blue) and maximum deviation (orange) between each string pathway iteration of **(j)** reduced and **(k)** oxidised states.

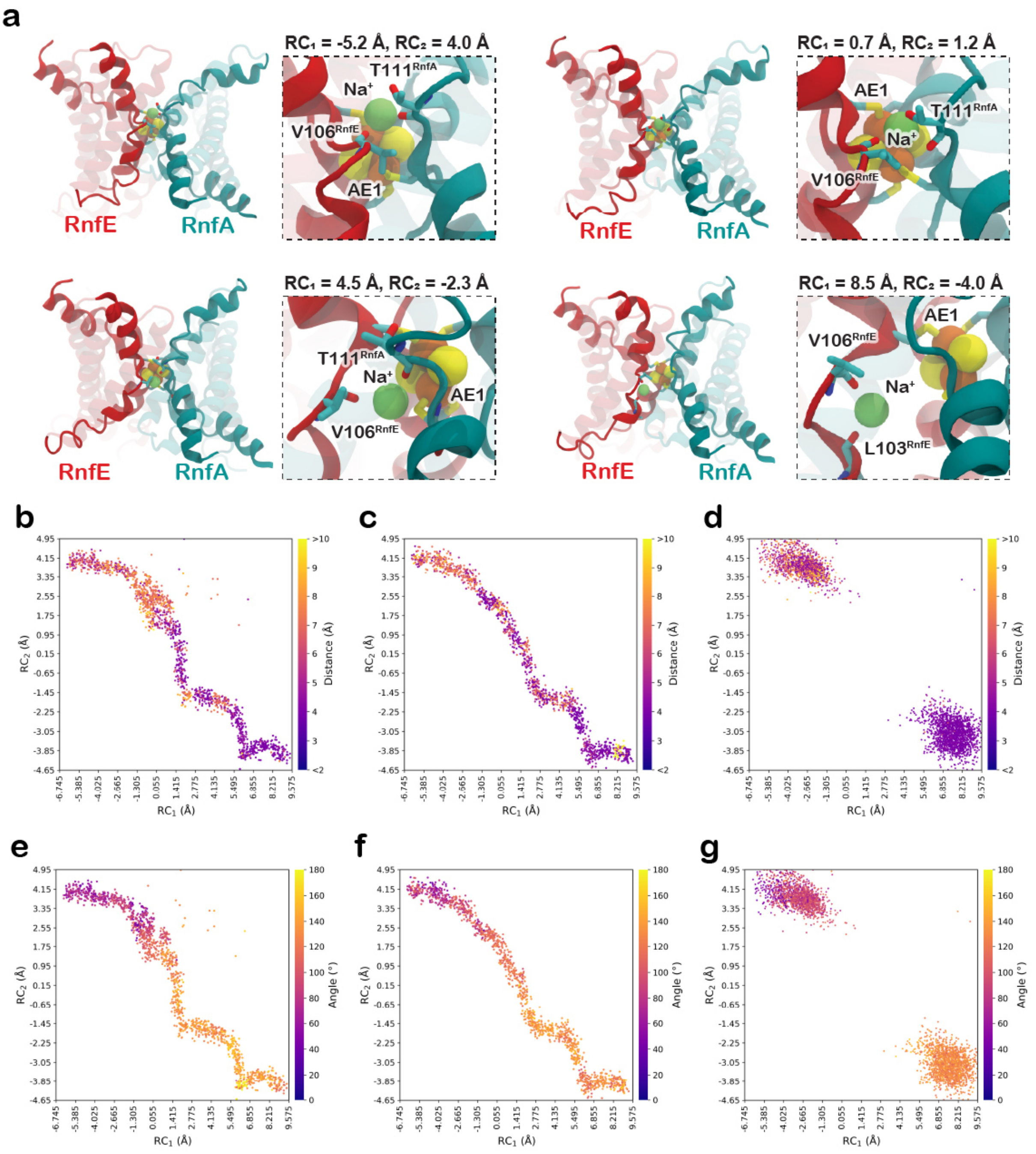

Supplementary Fig. 20 | Characterisation of structures and sampling along the string simulations. (a) Snapshots from the converged string simulation, showing the transport of a Na^+ ion from the intracellular (*top left*) to the extracellular side (*bottom right*) during the inward/outward transition. *Inset*: closeup of the Na^+ coordination by the backbone of residues $\text{T111}^{\text{RnfA}}$, $\text{V106}^{\text{RnfE}}$, and $\text{L103}^{\text{RnfE}}$. The reaction coordinates (RC_1 and RC_2) from each snapshot are given above the inset. **(b, c)** Distance of the $\text{E115}^{\text{RnfE}}$-$\text{R96}^{\text{RnfE}}$ ion pair during the string simulation for **(b)** the reduced and **(c)** oxidised states shows that the ion pair is open in the inward state, while it closes in the outward state. **(e, f)** Angle of the $\text{T111}^{\text{RnfA}}$ backbone carbonyl projected onto the Z-axis during the string simulation for the **(e)** reduced and **(f)** oxidised states. The carbonyl moiety flips as the RnfA/E subunit changes its conformation and transports the Na^+ ion. **(d)** $\text{E115}^{\text{RnfE}}$-$\text{R96}^{\text{RnfE}}$ ion pair distance and **(g)** $\text{T111}^{\text{RnfA}}$ backbone carbonyl angle from unbiased MD simulations (see Supplementary Table 6).

Supplementary References

1. Jo, S., Kim, T., Iyer, V. G. & Im, W. CHARMM-GUI: A web-based graphical user interface for CHARMM. *J. Comput. Chem.* **29**, 1859–1865 (2008).
2. Trabuco, L. G., Villa, E., Mitra, K., Frank, J. & Schulten, K. Flexible Fitting of Atomic Structures into Electron Microscopy Maps Using Molecular Dynamics. *Structure* **16**, 673–683 (2008).
3. Olsson, M. H. M., Søndergaard, C. R., Rostkowski, M. & Jensen, J. H. PROPKA3: Consistent Treatment of Internal and Surface Residues in Empirical p K_a Predictions. *J. Chem. Theory Comput.* **7**, 525–537 (2011).
4. Saura, P. & Kaila, V. R. I. Energetics and Dynamics of Proton-Coupled Electron Transfer in the NADH/FMN Site of Respiratory Complex I. *J. Am. Chem. Soc.* **141**, 5710–5719 (2019).
5. Gamiz-Hernandez, A. P., Jussupow, A., Johansson, M. P. & Kaila, V. R. I. Terminal Electron-Proton Transfer Dynamics in the Quinone Reduction of Respiratory Complex I. *J. Am. Chem. Soc.* **139**, 16282–16288 (2017).
6. Röpke, M. *et al.* Deactivation blocks proton pathways in the mitochondrial complex I. *Proc. Natl. Acad. Sci. U. S. A.* **118**, (2021).
7. Phillips, J. C. *et al.* Scalable molecular dynamics on CPU and GPU architectures with NAMD. *J. Chem. Phys.* **153**, 044130 (2020).
8. Humphrey, W., Dalke, A. & Schulten, K. VMD: Visual molecular dynamics. *J. Mol. Graph.* **14**, 33–38 (1996).
9. Gowers, R. *et al.* MDAnalysis: A Python Package for the Rapid Analysis of Molecular Dynamics Simulations. in 98–105 (Austin, Texas, 2016). doi:10.25080/Majora-629e541a-00e.
10. Fiorin, G., Klein, M. L. & Hénin, J. Using collective variables to drive molecular dynamics simulations. *Mol. Phys.* **111**, 3345–3362 (2013).
11. Finite Temperature String Method for the Study of Rare Events | The Journal of Physical Chemistry B. https://pubs.acs.org/doi/10.1021/jp0455430.
12. Riepl, D., Abou-Hamdan, A., Gellner, J., Sjöstrand, D., Högbom, M., von Ballmoos, C. & Kaila, V.R.I. Molecular principles of proton-coupled quinone reduction in the membrane-bound superoxide oxidase. *J. Am. Chem. Soc.* **147**, 6866-6879 (2025).
13. Grossfield, A. WHAM: the weighted histogram analysis method. *http://membrane.urmc.rochester.edu/wordpress/?page_id=126.*
14. Baker, N. A., Sept, D., Joseph, S., Holst, M. J. & McCammon, J. A. Electrostatics of nanosystems: Application to microtubules and the ribosome. *Proc. Natl. Acad. Sci.* **98**, 10037–10041 (2001).
15. Jurrus, E. *et al.* Improvements to the APBS biomolecular solvation software suite. *Protein Sci. Publ. Protein Soc.* **27**, 112–128 (2018).
16. Chovancova, E. *et al.* CAVER 3.0: A Tool for the Analysis of Transport Pathways in Dynamic Protein Structures. *PLoS Comput. Biol.* **8**, e1002708 (2012).
17. Magdziarz, T. *et al.* AQUA-DUCT 1.0: structural and functional analysis of macromolecules from an intramolecular voids perspective. *Bioinformatics* **36**, 2599–2601 (2020).
18. Schrödinger, LLC. The PyMOL Molecular Graphics System, Version 1.8. (2015).
19. Marcus, R. A. & Sutin, N. Electron transfers in chemistry and biology. *Biochim. Biophys. Acta BBA - Rev. Bioenerg.* **811**, 265–322 (1985).
20. Page, C. C., Moser, C. C., Chen, X. & Dutton, P. L. Natural engineering principles of electron tunnelling in biological oxidation–reduction. *Nature* **402**, 47–52 (1999).
21. Kaila, V. R. I., Johansson, M. P., Sundholm, D. & Wikström, M. Interheme electron tunneling in cytochrome *c* oxidase. *Proc. Natl. Acad. Sci.* **107**, 21470–21475 (2010).
22. Hoops, S. *et al.* COPASI—a COmplex PAthway SImulator. *Bioinformatics* **22**, 3067–3074 (2006).
23. Sievers, F. *et al.* Fast, scalable generation of high-quality protein multiple sequence alignments using Clustal Omega. *Mol. Syst. Biol.* **7**, 539 (2011).
24. Kaila, V. R. I. Long-range proton-coupled electron transfer in biological energy conversion: towards mechanistic understanding of respiratory complex I. *J R Soc Interface* **15**, (2017).
25. Punjani, A., Rubinstein, J. L., Fleet, D. J. & Brubaker, M. A. cryoSPARC: algorithms for rapid unsupervised cryo-EM structure determination. *Nat. Methods* **14**, 290–296 (2017).
26. Kishikawa, J. *et al.* Cryo-EM structures of Na+-pumping NADH-ubiquinone oxidoreductase from Vibrio cholerae. *Nat. Commun.* **13**, 4082 (2022).
27. Hau, J.-L. *et al.* Conformational coupling of redox-driven Na+-translocation in Vibrio cholerae NADH:quinone oxidoreductase. *Nat. Struct. Mol. Biol.* (2023) doi:10.1038/s41594-023-01099-0.
