# Supplementary material for: Molecular principles of redox-coupled sodium pumping of the ancient Rnf machinery
Source: Nat Commun. 2025 Mar 7;16:2302. doi: 10.1038/s41467-025-57375-8 (PMC11889175; doi:10.1038/s41467-025-57375-8)
Supplement: Supplementary file 2 — Description of Additional Supplementary Files [file 41467_2025_57375_MOESM2_ESM.pdf]

Description of Additional Supplementary Files

File name: Supplementary Movie 1
Description: Representation of the segmented cryoEM map of Rnf and its corresponding model.

File name: Supplementary Movie 2
Description: Sodium binding from the intracellular and extracellular sides.

File name: Supplementary Movie 3
Description: Inward/outward transition from MD simulations.

File name: Supplementary Movie 4
Description: Dominant normal modes from MD simulations of the NADH-reduced structures.

File name: Supplementary Movie 5
Description: Dominant normal modes from MD simulations of the Fd-reduced structures.

File name: Supplementary Movie 6
Description: Inward/outward pathway explored by SMD simulations.

File name: Supplementary Movie 7
Description: Free energy simulations of the inward/outward transition coupled to Na^+ transport.
