# Supplementary material for: Molecular principles of redox-coupled sodium pumping of the ancient Rnf machinery
Source: Nat Commun. 2025 Mar 7;16:2302. doi: 10.1038/s41467-025-57375-8 (PMC11889175; doi:10.1038/s41467-025-57375-8)
Supplement: Supplementary file 10 — Reporting Summary [file 41467_2025_57375_MOESM10_ESM.pdf]

nature portfolio

Corresponding author(s): Ville R. I. Kaila, Jan Schuller, Volker Müller

Last updated by author(s): Jan 15, 2025

Reporting Summary

Nature Portfolio wishes to improve the reproducibility of the work that we publish. This form provides structure for consistency and transparency in reporting. For further information on Nature Portfolio policies, see our Editorial Policies and the Editorial Policy Checklist.

Statistics

For all statistical analyses, confirm that the following items are present in the figure legend, table legend, main text, or Methods section.

n/a	Confirmed	
☐	☒	The exact sample size (*n*) for each experimental group/condition, given as a discrete number and unit of measurement
☐	☒	A statement on whether measurements were taken from distinct samples or whether the same sample was measured repeatedly
☒	☐	The statistical test(s) used AND whether they are one- or two-sided<br>*Only common tests should be described solely by name; describe more complex techniques in the Methods section.*
☒	☐	A description of all covariates tested
☒	☐	A description of any assumptions or corrections, such as tests of normality and adjustment for multiple comparisons
☐	☒	A full description of the statistical parameters including central tendency (e.g. means) or other basic estimates (e.g. regression coefficient) AND variation (e.g. standard deviation) or associated estimates of uncertainty (e.g. confidence intervals)
☒	☐	For null hypothesis testing, the test statistic (e.g. *F*, *t*, *r*) with confidence intervals, effect sizes, degrees of freedom and *P* value noted<br>*Give P values as exact values whenever suitable.*
☒	☐	For Bayesian analysis, information on the choice of priors and Markov chain Monte Carlo settings
☒	☐	For hierarchical and complex designs, identification of the appropriate level for tests and full reporting of outcomes
☒	☐	Estimates of effect sizes (e.g. Cohen's *d*, Pearson's *r*), indicating how they were calculated

Our web collection on statistics for biologists contains articles on many of the points above.

Software and code

Policy information about availability of computer code

Data collection	Size exclusion chromatography data was collected using UNICORN 5.31 software. WinASPECT 2.5.0.0 software was used for collecting photometric data. SerialEM and EPU software was used to evaluate EM micrographs.
Data analysis	Cryo electron microscopy data was analyzed with CryoSparc software v4.6.1. For analysis and image generation of protein structure, UCSF Chimera 1.16, UCSF ChimeraX 1.5 and PyMOL open source software was used. For model building and refinement, Coot and PHENIX software packages and tools were used. Prediction of structural models was performed using AlphaFold software. Quality assessment of the protein model was performed using PHENIX. GSFSC analysis was performed using CryoSparc. Size exclusion chromatography data was analyzed using UNICORN 5.31 software. WinASPECT 2.5.0.0 software was used for analyzing photometric data. The mass photometry was performed using OneMP mass photometer, and data was collected using AcquireMP.<br>MD simulations were performed with NAMD v2.14 and NAMD v3.0.<br>Electrostatic calculations were performed with APBS v3.4.1. Kinetic model with COPASI. For analysis and visualization, VMD v.1.9.3 and MDAnalysis were used. Cavity analysis was performed with CAVER 3.0. Multiple sequence alignment was performed with ClustalΩ, and the sequences were downloaded from Uniprot.

For manuscripts utilizing custom algorithms or software that are central to the research but not yet described in published literature, software must be made available to editors and reviewers. We strongly encourage code deposition in a community repository (e.g. GitHub). See the Nature Portfolio guidelines for submitting code & software for further information.

Data

Policy information about availability of data

All manuscripts must include a data availability statement. This statement should provide the following information, where applicable:

- Accession codes, unique identifiers, or web links for publicly available datasets
- A description of any restrictions on data availability
- For clinical datasets or third party data, please ensure that the statement adheres to our policy

Cryo-EM maps are available in the Electron Microscopy Data Bank; Rnf treated with NADH (19915), Rnf with reduced Fd state-1 consensus map (19919), Rnf with reduced Fd state-2 B8 closer to membrane (19916), and Rnf apo (19920). The atomic models of Rnf complex are available in the Protein Data Bank; Rnf treated with NADH (9ERI), Rnf with reduced Fd state-1 consensus map (9ERK), Rnf with reduced Fd state-2 B8 closer to membrane (9ERJ), and Rnf apo (9ERL). Structural and sequence data used for comparison with Rnf subunits are available in the Protein Data Bank (PDB – 7XK3, Nqr complex). Strains and plasmids generated in this study are available from the corresponding authors upon request. Force field parameters and MD simulation data are available in the zenodo repository (10.5281/zenodo.10974499).

Research involving human participants, their data, or biological material

Policy information about studies with human participants or human data. See also policy information about sex, gender (identity/presentation), and sexual orientation and race, ethnicity and racism.

Reporting on sex and gender	Not applicable
Reporting on race, ethnicity, or other socially relevant groupings	Not applicable
Population characteristics	Not applicable
Recruitment	Not applicable
Ethics oversight	Not applicable

Note that full information on the approval of the study protocol must also be provided in the manuscript.

Field-specific reporting

Please select the one below that is the best fit for your research. If you are not sure, read the appropriate sections before making your selection.

[X] Life sciences [ ] Behavioural & social sciences [ ] Ecological, evolutionary & environmental sciences

For a reference copy of the document with all sections, see nature.com/documents/nr-reporting-summary-flat.pdf

Life sciences study design

All studies must disclose on these points even when the disclosure is negative.

Sample size

Sample size was not predetermined by statistical methods but sample sizes of n=3 or more were used for in vitro analysis to provide data sets that enable statistics for the relevant experiments. The in vitro experiments are tried-and-tested procedures that have been successfully performed in the author´s laboratories day-to-day for many years. The sample sizes were diligently chosen and accurately illustrate the differences throughout the various conditions.

For cryo-EM, multiple data set containing 20-50K micrographs were acquired in order to have a sufficient number of Rnf particles, which resulted in a 3D reconstruction with a resolution of 2.5-3.3 Angstrom. The achieved resolution was enough to model the protein structure at the residue level.

MD simulations: MD trajectories were run for 100-700 ns each, with a total of 8300 ns

Data exclusions

For enzymatic assays of strictly anaerobic enzymes, oxygen contamination cannot always be excluded and leads to a significant reduction of enzymatic activity. If a measurement was obviously differing from the other replicates, this measurement was excluded from the data set. For cryo-EM, monomeric Rnf complex particles were picked for determination of the high resolution structure. Other particles were excluded from the analysis. No data were excluded from biochemical, biophysical or computational experiments.

Replication

Enzymatic assays were performed in biological triplicates with three technical repetitions each. Sample separation via polyacrylamide gels or size exclusion chromatography was performed at least in two independent biological repetitions, respectively. For cryo-EM analysis of 20-50K micrographs from each data set were analyzed. All assays could be successfully replicated.
MD simulations: 11 MD trajectories were performed in duplicates.

Randomization

Resolution Determination was carried out with two independent half sets randomly selected by the CryoSparc processing software according

Randomization	to the Gold-Standard FSC procedure. For all other experiments, randomization was not applicable, since in microbiological assays all parameters are tightly controlled and therefore covariates are not relevant to our study.
Blinding	Blinding was not relevant to our study since no higher order species were used in this study. Moreover we have repeated experiments in biological triplicates with 3 technical replicates, and all assays were reproducible.

Reporting for specific materials, systems and methods

We require information from authors about some types of materials, experimental systems and methods used in many studies. Here, indicate whether each material, system or method listed is relevant to your study. If you are not sure if a list item applies to your research, read the appropriate section before selecting a response.

Materials & experimental systems

n/a	Involved in the study
☒	☐ Antibodies
☒	☐ Eukaryotic cell lines
☒	☐ Palaeontology and archaeology
☒	☐ Animals and other organisms
☒	☐ Clinical data
☒	☐ Dual use research of concern
☒	☐ Plants

Methods

n/a	Involved in the study
☒	☐ ChIP-seq
☒	☐ Flow cytometry
☒	☐ MRI-based neuroimaging

Plants

Seed stocks	Not applicable
Novel plant genotypes	Not applicable
Authentication	Not applicable
